# Cluster Randomised Trials in Cochrane Reviews: Evaluation of Methodological and Reporting Practice

**DOI:** 10.1371/journal.pone.0151818

**Published:** 2016-03-16

**Authors:** Marty Richardson, Paul Garner, Sarah Donegan

**Affiliations:** 1 Centre for Evidence Synthesis in Global Health, Department of Clinical Sciences, Liverpool School of Tropical Medicine, Liverpool, United Kingdom; 2 Department of Biostatistics, Block F Waterhouse Building, University of Liverpool, Liverpool, United Kingdom; University Hospital Oldenburg, GERMANY

## Abstract

**Objective:**

Systematic reviews can include cluster-randomised controlled trials (C-RCTs), which require different analysis compared with standard individual-randomised controlled trials. However, it is not known whether review authors follow the methodological and reporting guidance when including these trials. The aim of this study was to assess the methodological and reporting practice of Cochrane reviews that included C-RCTs against criteria developed from existing guidance.

**Methods:**

Criteria were developed, based on methodological literature and personal experience supervising review production and quality. Criteria were grouped into four themes: identifying, reporting, assessing risk of bias, and analysing C-RCTs. The Cochrane Database of Systematic Reviews was searched (2^nd^ December 2013), and the 50 most recent reviews that included C-RCTs were retrieved. Each review was then assessed using the criteria.

**Results:**

The 50 reviews we identified were published by 26 Cochrane Review Groups between June 2013 and November 2013. For identifying C-RCTs, only 56% identified that C-RCTs were eligible for inclusion in the review in the eligibility criteria. For reporting C-RCTs, only eight (24%) of the 33 reviews reported the method of cluster adjustment for their included C-RCTs. For assessing risk of bias, only one review assessed all five C-RCT-specific risk-of-bias criteria. For analysing C-RCTs, of the 27 reviews that presented unadjusted data, only nine (33%) provided a warning that confidence intervals may be artificially narrow. Of the 34 reviews that reported data from unadjusted C-RCTs, only 13 (38%) excluded the unadjusted results from the meta-analyses.

**Conclusions:**

The methodological and reporting practices in Cochrane reviews incorporating C-RCTs could be greatly improved, particularly with regard to analyses. Criteria developed as part of the current study could be used by review authors or editors to identify errors and improve the quality of published systematic reviews incorporating C-RCTs.

## Introduction

Systematic reviews summarise existing studies of interventions for a particular disease. Cochrane reviews are high-quality systematic reviews of primary research in human health care and health policy, and are conducted using standard methods by review groups within Cochrane [1]. Randomised controlled trials are considered to be the highest quality of primary research study design and are therefore often included in such reviews. Individual-randomised trials (I-RCTs) and/or cluster-randomised trials (C-RCTs) can be included.

In an I-RCT, individual participants are randomly allocated to intervention groups. However, sometimes it is impractical, even impossible, to randomise individuals but it may be feasible to randomise clusters of individuals (e.g. schools, communities or clinics) to intervention groups [2]. Therefore, in such trials, the unit of randomisation is the cluster rather than the individual. For example, to evaluate the effect of insecticidal spraying of a household on malaria prevalence, it would be impossible to randomise individuals to spraying or no spraying when more than one person lives in the same household because the whole household is usually sprayed; however, households could be randomised to spraying or no spraying. Consequently, C-RCTs are important in evaluating a variety of public health and health service interventions. Furthermore, C-RCTs can also be used when multiple outcome measurements are taken on the same individual (e.g. to evaluate the effectiveness of a topical cream for a skin condition, one measurement could be taken on each arm of the same individual); in such cases, individuals are randomised to interventions and each individual is considered to be a cluster.

The Cochrane Handbook [3] and other methodological publications [4–6] provide guidance regarding the inclusion of C-RCTs in reviews. This guidance includes details on how to assess the risk of bias, extract data, and analyse C-RCTs. If review authors do not follow the guidance, but instead, analyse C-RCTs in the same way as I-RCTs, the confidence interval (CI) for the treatment effect would be artificially narrow because clustering would not be taken into account. Interpreting such analyses that are not adjusted for clustering may lead to false conclusions being drawn from the review and result in patients being treated with inferior interventions. Review authors may be able to adjust treatment effect estimates for clustering themselves using estimates of the average cluster size and intracluster correlation coefficient (ICC), which quantifies the extent to which data from observations from the same cluster are correlated [3].

This report introduces assessment criteria that were developed based on the published guidance, and which were used to examine the methodological and reporting quality of Cochrane reviews that include C-RCTs. Specifically, this study assesses whether the following details are considered and/or reported in systematic reviews: C-RCTs are identified throughout the review; general cluster information is reported; risk of bias is assessed appropriately; and analyses are carried out correctly. The study also ascertained how often reviews incorrectly analyse C-RCTs in the same way as I-RCTs. This research updates and extends the previously published review by Laopaiboon *et al*. [6], which examined synthesis approaches of meta-analyses involving C-RCTs.

## Methods

### The Assessment Criteria

Criteria were developed to assess the quality of each review that included C-RCTs. The criteria were categorised under four themes: identifying C-RCTs, reporting C-RCTs, assessing risk of bias of C-RCTs, and analysing C-RCTs. Table 1 displays the criteria for each theme. Each criterion took the form of a question that could be answered “Yes” (representing current best practice), “No” (does not fulfil this criterion), or “N/A” (when it was not possible for the review to meet this criterion).

**Table 1:** Assessment criteria and results.

Criterion	Yes	
	%	No. of reviews/total no. of reviews
**Identifying C-RCTs**		
1. Abstract: are C-RCTs mentioned?	48	24/50
2. Eligibility criteria (‘Types of studies’): are C-RCTs mentioned?	56	28/50
3. Methods: are methods specific to C-RCTs described?	84	42/50
4. Description of studies results text: is each C-RCT identified?	68	34/50
5. Characteristics of included studies results table: are C-RCTs identified?	94	47/50
6. Assessment of risk of bias results tables: is each C-RCT identified?	48	24/50
7. Is each C-RCT identified in the efficacy results (i.e. text, tables, or forest plots)?	36	18/50
**Reporting C-RCTs**		
8. Is the unit of randomisation reported for each C-RCT?	90	44/49^d^
9. Is the study design (i.e. matched pairs, stratified) reported for each C-RCT?	69	34/49^d^
10. Is it reported whether the trial is adjusted or unadjusted for clustering for each C-RCT for each outcome?	59	29/49^d^
11. Is the method of cluster adjustment reported for each C-RCT for each outcome?	24	8/33^e^
12. Is the ICC reported for each C-RCT for each outcome?	30	7/23^f^
13. Is the average cluster size reported for each C-RCT?	48	22/46^g^
**Assessing risk of bias of C-RCTs**		
14. Is risk of recruitment bias assessed for each C-RCT?	10	5/50
15. Is risk of baseline imbalances assessed for each C-RCT?	52	26/50
16. Is risk of loss of clusters and individuals assessed for each C-RCT?	24	12/50
17. Is risk of incorrect analysis assessed for each C-RCT?	26	13/50
18. Is risk of non-comparability with I-RCTs assessed for each C-RCT for each outcome?	2	1/45^h^
**Analysing C-RCTs**^a^		
19. Is it stated whether results for each C-RCT for each outcome are adjusted (i.e. in the text, tables, or forest plots)?	26	13/50
20. Is there a warning that CIs may be artificially narrow whenever unadjusted results are presented?	33	9/27*
21. Are all unadjusted results from C-RCTs excluded from the meta-analysis?	38	13/34^†^
22. Are all unadjusted results adjusted using average cluster size and ICC extracted from the trial report?^b^	22	6/27^‡^
23. If an ICC is estimated, are sensitivity analyses carried out using different ICC estimates?	50	5/10^§^
24. Are data correctly extracted for each outcome for each C-RCT that adjust for clustering?^c^	68	21/31^‖^
25. Are C-RCTs and I-RCTs sub-grouped for each outcome in the text, tables or forest plots?	13	5/40^¶^
26. Are C-RCTs sub-grouped by unit of randomisation for each outcome in the text, tables, or forest plots?	0	0/22^£^

^a^ A review met these criteria 19–26 if the review satisfied the criterion for every outcome in the review

^b^ A review met this criterion if the review stated that the review authors used this method in the methods or results

^c^ A review met this criterion if the review stated that the review authors adjusted for clustering themselves in the methods or results

^d^ 1 review excluded as none of the trial reports for the included C-RCTs could be obtained via inter-library loans

^e^ 17 reviews excluded (1 review: none of the trial reports for the included C-RCTs could be obtained; 4 reviews: as all trials did not report method of adjustment; 12 reviews: as all trials were unadjusted)

^f^ 27 reviews excluded (1 review: none of the trial reports for the included C-RCTs could be obtained; 23 reviews: all trials did not report ICC, 3 reviews: some trials did not report ICC and some trial reports were unavailable)

^g^ 4 reviews excluded (1 review: none of the trial reports for the included C-RCTs could be obtained; 3 reviews: all trials did not report average cluster size)

^h^ 5 reviews excluded that only included C-RCTs

* Criteria was applicable for 27 reviews that presented unadjusted results

^†^ Criteria was applicable for 34 reviews where data from unadjusted C-RCTs was eligible for inclusion in the conducted meta-analyses

^‡^ Criteria was applicable for 27 reviews that included trials with unadjusted data, and that did not state that it would not be possible to adjust data themselves

^§^ Criteria was applicable for 10 reviews that estimated an ICC to adjust cluster data

^‖^ Criteria was applicable for 31 reviews where included trials reported adjusted data

^¶^ Criteria was applicable for 40 reviews that included both C-RCTs and I-RCTs in the same analysis

^£^ Criteria was applicable to 22 reviews that included C-RCTs with different units of randomisation in the same analysis

CI: confidence interval; C-RCT: cluster-randomised controlled trial; ICC: intracluster correlation coefficient; I-RCT: individually-randomised controlled trial

The criteria were based on recommendations for review authors given in the Cochrane Handbook [3] and methodological publications [2, 4–6] and our own experience as editors of a Cochrane Review Group. The CONSORT statement for C-RCTs [2], which provides reporting guidelines for C-RCT reports, was also consulted.

### Eligibility Criteria

The 50 most recent Cochrane intervention reviews (new or updated) that reported the term “cluster” and/or “community” in the review (or references of included studies) and that included at least one C-RCT were eligible for inclusion in the current study. The presence of at least one C-RCT in the review was verified by obtaining the original trial articles for which the term “cluster” and “community” was used in the review. Review protocols and withdrawn reviews were excluded. Fifty reviews were considered sufficient to obtain a picture of current practice while keeping the study manageable. Only Cochrane Reviews were included because explicit guidance is available and promoted to review authors in the Cochrane handbook [3]; therefore, all review authors were expected to have followed this guidance (i.e. criteria 14–24 in Table 1), and it was hoped that some may also have met the additional criteria identified in the current study, which are believed to be important when including C-RCTs in Cochrane reviews.

### Search Strategy

Reviews were sought via the Cochrane Database of Systematic Reviews (29th November, 2013) using the search terms “cluster” OR “community”.

### Review Selection

The full text was obtained for each review identified by the search. One author (MR) assessed the eligibility of reviews using a pre-prepared form. If eligibility was unclear, the review authors were contacted for more information.

### Data Extraction

One author (MR) extracted data from both the reviews and their included C-RCTs using two pre-piloted forms (one for review characteristics, one for trial characteristics). A second author (SD) independently extracted data for a sample of 10 reviews (20%); any differences in opinion between the two authors were resolved through discussion. The full trial report was retrieved for every C-RCT trial that was labelled as “community” or “cluster” in the review, and trials with “cluster” or “community” in the title. The original trial reports for each C-RCT were obtained by using the University of Liverpool electronic library and through inter-library loans. Data were extracted from the C-RCTs in order to assess the reviews using the criteria (e.g. if the C-RCT reported cluster-adjusted results, the review was also expected to report the adjusted results, whereas the review was not expected to report adjusted results if the C-RCT trial had not reported these data).

Information was extracted regarding review characteristics, and the C-RCT details (identifying C-RCTs, reporting C-RCTs, assessing risk of bias of C-RCTs, and analysing C-RCTs). See S1–S5 Tables for the type of information that was extracted. Criteria were assessed for all outcomes in the included reviews where necessary; for example, if a review did not perform analyses correctly for one outcome but performed analyses correctly for other outcomes, the review was assessed to have not met the criteria.

### Data Analysis

The characteristics of each review and C-RCTs were tabulated. Each review was assessed by MR against the proposed criteria using pre-piloted assessment forms. It was possible for one review to meet a criterion for some included C-RCTs but not meet the same criterion for other C-RCTs. For example for criterion 8, unit of randomisation may be reported for some C-RCTs but not for others. Therefore, the percentage of reviews meeting each criterion for all included C-RCTs was calculated and presented. For criteria 12, 18, 19–26, it was possible for a particular review to meet the criteria for one outcome but not for other outcomes; for these criteria, a review was judged to satisfy the criterion if the review satisfied the criterion for every outcome in the review. Reviews for which a criterion was not applicable were excluded from the calculation. The assessment result for each criterion for each review were tabulated.

## Results

A total of 276 reviews were assessed for eligibility, and 50 reviews were included [7–56]. S1 Table gives details of the included trials, participants, interventions and outcomes of each review. The 50 reviews included 1170 trials in total; the median number of trials included was 18 (interquartile range [IQR] 9–29, range 1–77).

A total of 201 C-RCTs were included in the included reviews, and references for these C-RCTs are provided in S1 File. The median number of C-RCTs included was 3 (IQR 1–6, range 1–23). The percentage of included C-RCTs ranged from 2% to 100%.

Forty-five reviews (90%) included both C-RCTs and I-RCTs; five reviews (10%) included C-RCTs alone. Forty-six reviews (92%) included C-RCTs that randomised groups of individuals to interventions, and four reviews (8%) included C-RCTs that randomised individuals to interventions, with each individual considered to be a cluster of observations (i.e. parts of the body, multiple pregnancies, or episodes of a condition).

**Fig 1:**
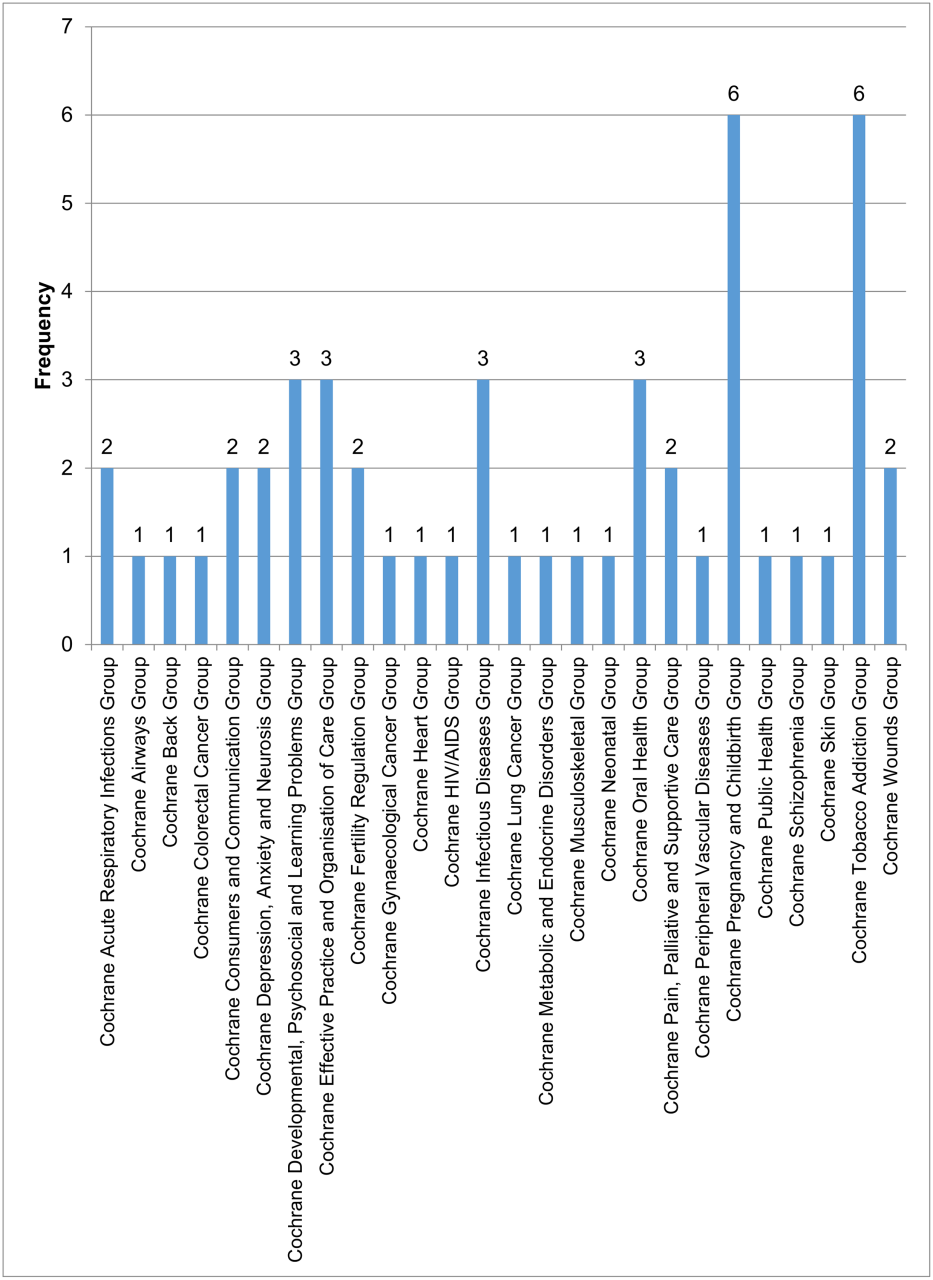
Number of included reviews by Cochrane Review Group.

The included reviews varied considerably in terms of disease area (S1 Table). Fig 1 shows the number of included reviews by Cochrane Review Group. Reviews were published by 26 Cochrane Review Groups with each group including between one and six reviews.

Interventions compared included: non-pharmacological (21 reviews); drugs (six reviews); screening (five reviews); training/education (four reviews); counselling/advice (four reviews); behavioural (two reviews); techniques (two reviews); psychosocial (one review); vaccination (one review); drugs/psychosocial/non-pharmacological (one review), drugs/non-pharmacological (one review), drugs/psychosocial (one review), drugs/psychosocial/behavioural (one review). A variety of outcomes were included in the reviews (S1 Table).

Table 1 shows the percentage of reviews that met each criterion. Assessment results for each criterion for each review are provided in S2 Table for “Identifying C-RCTs”, S3 Table for “Reporting C-RCTs”, S4 Table for “Assessing risk of bias of C-RCTs”, and S5 Table for “Analysing C-RCTs”.

### Identifying C-RCTs

About half of the abstracts of the reviews reported C-RCTs (24 reviews, 48%), and a little more than half were explicit that C-RCTs were eligible for inclusion (28 reviews, 56%). Most reviews described methods for analysis of C-RCTs (42 reviews, 84%). In results, 68% of reviews (34 reviews) identified C-RCTs in the description of the included studies (the “Description of studies” text), and most reviews (47 reviews, 94%) identified C-RCTs in the detailed “Characteristics of included studies” table. Only 24 reviews (48%) referred to C-RCTs in the risk of bias results tables, and fewer still (18 reviews, 36%) labelled C-RCTs in the efficacy results (i.e. text, tables, or forest plots).

### Reporting C-RCTs

One review was excluded from this analysis because none of the included C-RCT reports could be obtained. Of the remaining 49 reviews, systematic reporting varied across the criteria: 44 (90%) reported the unit of randomisation; 34 (69%) reported the study design; 22 (48%) reported the average cluster size; 29 (59%) reported whether the data were cluster-adjusted; eight (24%) reported the analysis method; and seven (30%) reported the ICC for each outcome of interest in each C-RCT.

The percentages of reviews that reported these characteristics for at least one of the included C-RCTs (as opposed to all included C-RCTs, as presented in Table 1) was also calculated. These results are provided in S6 Table.

### Assessing Risk of Bias of C-RCTs

More than half of the reviews (26 reviews, 52%) assessed the risk of baseline imbalances, whereas only one review (2%) assessed the risk of non-comparability with RCTs. The assessment of comparability between I-RCTs and C-RCTs was not applicable to five reviews that did not include I-RCTs. A small percentage of reviews assessed the risk of recruitment bias (10%), the risk of loss of clusters and individuals (24%), and the risk of incorrect analysis (26%).

Only one review assessed all risk of bias criteria.

### Analysing C-RCTS

Only 26% of reviews (13 reviews) stated whether all C-RCT results were adjusted or unadjusted in the forest plots, text or tables.

The analysis of unadjusted data from C-RCTs was poor. Of the 27 reviews that presented unadjusted data, only nine (33%) provided a warning to say that CIs may be artificially narrow. Of the 34 reviews that reported unadjusted data, only 13 (38%) excluded the unadjusted results from the meta-analyses.

Of 27 reviews that included trials with unadjusted data that did not state that it would not be possible to adjust data themselves, six (22%) adjusted unadjusted cluster data using the average cluster size and ICC as reported in the C-RCT trial reports. In the case of 10 reviews that estimated an ICC (i.e. from similar trials, or elsewhere in the literature) to adjust cluster data, five (50%) carried out sensitivity analyses to investigate the effect of estimating ICC.

A total of 31 reviews included trials that adjusted for clustering, of which 21 (68%) correctly extracted adjusted data.

Of the 40 reviews that included both C-RCTs and I-RCTs in the same meta-analysis, only five reviews (13%) subgrouped the data according to study design to enable correct interpretation or investigation of heterogeneity. Furthermore, of 22 reviews that included C-RCTs with different units of randomisation in the same meta-analysis, no review subgrouped the data according to unit of randomisation.

## Discussion

In the current study, assessment criteria were devised and used to evaluate how well Cochrane reviews that included C-RCTs were carried out in practice. Findings showed that reviews could be substantially improved in terms of methodology and reporting.

The results of this study suggested that there is room for improvement in the identifying of C-RCTs for each of the assessed criteria. Perhaps the most concerning findings were that only 56% of reviews specified that C-RCTs were eligible for inclusion in the eligibility criteria (28 reviews), and that eight reviews did not describe any methods specific to C-RCTs (16%).

In order to ensure that readers of Cochrane review understand the study design of included C-RCTs and are able to interpret results correctly, review authors ought to report several details about the C-RCT. The results of the current study suggested that Cochrane review authors are unaware of the importance of reporting these details about included C-RCTs; reporting standards were low across all criteria, with the exception of “unit of randomisation”. Only 59% of the reviews reported whether C-RCT results were adjusted for clustering, and therefore readers of these reviews would be unable to assess whether the data had been analysed correctly.

The five additional risk-of-bias criteria for C-RCTs are listed in the Cochrane Handbook [3]. Review authors ought to apply these assessments to ensure that the quality of each C-RCT trial has been adequately assessed; this is in addition to the criteria listed for assessing risk of bias for I-RCTs [3]. Only one review assessed risk of bias for all additional criteria for included C-RCTs; hence, conclusions for the remaining reviews may have been drawn based on low quality evidence from studies that were not adequately appraised.

The importance of performing appropriate analyses for C-RCTs is crucial because errors in the analyses can affect results and influence healthcare decisions. Only 26% of included reviews stated whether C-RCT results were adjusted, and only 38% of the reviews excluded unadjusted results from the meta-analyses. When unadjusted results are presented without indicating that no adjustments for clustering have been performed, CIs are too narrow and these incorrect results may influence conclusions drawn from the review. When confidence intervals are too narrow for C-RCTs, the overall pooled effect is more precise than it should be and this may lead to incorrect conclusions (e.g. a significant difference may be found where none exists). Furthermore, if confidence intervals are too narrow for C-RCTs, this also implies that inverse variance weights for these trials are overestimated, and thus the C-RCTs obtain more weight in the meta-analysis than they should. This may cause bias in favour of the results generated by C-RCTs.

We believe that the current findings indicate that review authors do not understand or are not aware of appropriate methods and reporting in reviews including C-RCTs. The findings corroborate with research conducted in 2003; a study of 25 meta-analyses involving C-RCTs found that half (13/25) used inappropriate analysis methods that ignored the effect of clustering (i.e. review authors treated the included C-RCTs as though they were I-RCTs), and pooled these invalid results in meta-analyses [6]. In the current study, only 38% of reviews excluded unadjusted results from C-RCTs in meta-analyses, suggesting that practice has not greatly improved since 2003. Furthermore, another study reviewed all reviews of the Cochrane Infectious Diseases Group without the use of search terms, and found that results were comparable to those in the current study [57]. Review authors often used the same risk-of-bias assessment components for the assessment of C-RCTs and I-RCTs (86%, *n* = 92); this corroborates with the findings of this review (only one review applied all five cluster-specific risk-of-bias criteria), suggesting that very few authors perform risk-of-bias assessments that are specific to C-RCTs. Issues with incorrect analyses were also highlighted in the review of Cochrane Infectious Diseases Group reviews; for example, it was found that 57% of reviews (*n* = 92) combined non-adjusted C-RCTs with I-RCTs in the meta-analysis. [57]

### Limitations and Further Work

The main limitation of this research is that only Cochrane reviews were included. Yet, the criteria are certainly applicable to other types of reviews, such as health technology assessment reviews and reviews published in journals. However, the results in the current study may not be generalisable to other types of reviews because guidance regarding the inclusion of C-RCTs is given in the Cochrane handbook and extra support is provided by Cochrane editorial teams. As further work, this project could be extended to assess the inclusion of C-RCTs in other types of reviews.

A second limitation of the current study is that only reviews that used the term “cluster” or “community” were included; therefore, any reviews that included C-RCTs but did not use these specific terms would not have been included. Identifying such reviews would involve obtaining the published article for each included trial in each review and would not be feasible. Such reviews may have been of poorer quality; therefore, the results reported in this article may be an underestimation of the methodological and reporting deficiencies identified in this study.

A further limitation of this study is the search date; the last search was carried out in November 2013 and the included reviews were published between June and December 2013. Although, many Cochrane reviews have been published since this date, they are unlikely to differ greatly from those included in the current study because, to the authors’ knowledge, no updated or new guidance has been published or presented at conferences.

In some respects, the quality of a review that includes C-RCTs is limited by the methodological and reporting quality of the included C-RCTs. For example, if a study does not adjust data for clustering, and does not report an ICC and average cluster size, it is difficult for review authors to obtain an estimate of treatment effect which takes the effects of clustering into consideration. The current study highlights that flaws exist in published C-RCTS, with many of the included C-RCTs not reporting key information, and not adjusting analyses for clustering. This could be due to trial authors not following the Consort statement for C-RCTs [2], or statistical peer reviewers not identifying lack of adjusted analyses, or even included trials being published before the Consort statement was published. Further work could explore how often trials are poorly reported and why this is the case.

### Recommendations to Authors of Reviews

Table 2 provides a checklist that we recommend review authors use when including C-RCTs in reviews. The checklist is applicable to any systematic review incorporating C-RCTs. Review authors can use the checklist to carry out their review and editorial teams and users of reviews can use the checklist to check reviews for errors.

**Table 2:** Checklist for reviews including C-RCTs.

Criterion	Y/N
**Identifying C-RCTs**	
1. Abstract: are C-RCTs mentioned?	
2. Eligibility criteria (‘Types of studies’): are C-RCTs mentioned?	
3. Methods: are methods specific to C-RCTs described?^a^	
4. Description of studies results text: is each C-RCT identified?	
5. Characteristics of included studies results table: are C-RCTs identified?	
6. Assessment of risk of bias results tables: is each C-RCT identified?	
7. Is each C-RCT identified in the efficacy results (i.e. text, tables, or forest plots)?	
**Reporting C-RCTs**^b^	
8. Is the unit of randomisation reported for each C-RCT?	
9. Is the study design (i.e. matched pairs, stratified) reported for each C-RCT?	
10. Is it reported whether the trial is adjusted or unadjusted for clustering for each C-RCT for each outcome?	
11. Is the method of cluster adjustment reported for each C-RCT for each outcome?	
12. Is the ICC reported for each C-RCT for each outcome?	
13. Is the average cluster size reported for each C-RCT?	
**Assessing risk of bias of C-RCTs**	
14. Is risk of recruitment bias assessed for each C-RCT?	
15. Is risk of baseline imbalances assessed for each C-RCT?	
16. Is risk of loss of clusters and individuals assessed for each C-RCT?	
17. Is risk of incorrect analysis assessed for each C-RCT?	
18. Is risk of non-comparability with I-RCTs assessed for each C-RCT for each outcome?	
**Analysing C-RCTs**	
19. Is it stated whether results for each C-RCT for each outcome are adjusted (i.e. in the text, tables, or forest plots)?	
20. Is there a warning that CIs may be artificially narrow whenever unadjusted results are presented?	
21. Are all unadjusted results from C-RCTs excluded from the meta-analysis?	
22. Are all unadjusted results adjusted using average cluster size and ICC extracted from the trial report?	
23. If an ICC is estimated, are sensitivity analyses carried out using different ICC estimates?	
24. Are data correctly extracted for each outcome for each C-RCT that adjust for clustering?	
25. Are C-RCTs and I-RCTs sub-grouped for each outcome in the text, tables or forest plots?^c^	
26. Are C-RCTs sub-grouped by unit of randomisation for each outcome in the text, tables, or forest plots?^c^	

^a^Data extraction, analysis and risk of bias methods specific to C-RCTs must be reported

^b^Data that are not reported in the published article could be sought from trial investigators. Report absences of key information in the original trial reports, and if necessary, comment on the possible implications of this information being missing

^c^Subgroup analyses can be performed to explore heterogeneity between results from C-RCTs and I-RCTs or between results from C-RCTs with different units of randomisation [4, 5]

CI: confidence interval; C-RCT: cluster-randomised controlled trial; ICC: intracluster correlation coefficient; I-RCT: individually-randomised controlled trial

### Conclusions

It is clear that a consolidated and comprehensive set of guidelines is needed that address the different aspects of including C-RCTs in systematic reviews to help improve the quality of published reviews including C-RCTs. An extension to existing systematic review and meta-analysis guidelines, such as the PRISMA statement [58], would meet this need; the criteria used in the current study could contribute to these extended guidelines. It is hoped that the current study will promote methods for including C-RCTs in reviews and help to improve the quality of future reviews.

## Supporting Information

S1 File References for included C-RCTs in each review. (DOCX)

S2 File PRISMA Checklist. (DOC)

S1 Table Summary of included reviews. (DOCX)

S2 Table Assessment of “Identifying C-RCTs”. (DOCX)

S3 Table Assessment of “Reporting C-RCTs”. (DOCX)

S4 Table Assessment of “Assessing risk of bias of C-RCTs”. (DOCX)

S5 Table Assessment of “Analysing C-RCTs”. (DOCX)

S6 Table Additional results for “Reporting C-RCTs”. (DOCX)
