# Supplementary material for: Cluster Randomised Trials in Cochrane Reviews: Evaluation of Methodological and Reporting Practice
Source: PLoS One. 2016 Mar 16;11(3):e0151818. doi: 10.1371/journal.pone.0151818 (PMC4794236; doi:10.1371/journal.pone.0151818)
Supplement: S1 File — (DOCX) [file pone.0151818.s001.docx]

Supplementary File 1. References for included C-RCTs in each review

| Review | Cochrane Group | Trial |
| --- | --- | --- |
| Antibiotics for preventing meningococcal infections | Cochrane Acute Respiratory Infections Group | Blakebrough and Gilles (1) |
|  |  | Cuevas, Kazembe (2) |
|  |  | Guttler, Counts (3) |
|  |  | Munford, Sussuarana de Vasconcelos (4) |
|  |  | Schwartz (5) |
| Influenza vaccination for healthcare workers who care for people aged 60 or older living in long-term care institutions | Cochrane Acute Respiratory Infections Group | Carman, Elder (6) |
|  |  | Potter, Stott (7) |
|  |  | Lemaitre, Meret (8) |
| Integrated disease management interventions for patients with chronic obstructive pulmonary disease | Cochrane Airways Group | Rea, McAuley (9) |
|  |  | Wood-Baker, McGlone (10) |
| Physical conditioning as part of a return to work strategy to reduce sickness absence for workers with back pain | Cochrane Back Group | Bethge, Herbold (11) |
|  |  | Loisel, Abenhaim (12) |
| Flexible sigmoidoscopy versus faecal occult blood testing for colorectal cancer screening in asymptomatic individuals | Cochrane Colorectal Cancer Group | Segnan, Armaroli (13) |
| Mass media interventions for reducing mental health-related stigma | Cochrane Consumers and Communication Group | Coleman (14) |
|  |  | Penn, Chamberlin (15) |
|  |  | Yoshida (NTR) |
| Interventions to promote informed consent for patients undergoing surgical and other invasive healthcare procedures | Cochrane Consumers and Communication Group | Solberg, Asche (16) |
|  |  | Paci, Barneschi (17) |
| Enhanced care by generalists for functional somatic symptoms and disorders in primary care | Cochrane Depression, Anxiety and Neurosis Group | Blankenstein (18) |
|  |  | Larisch, Schweickhardt (19) |
|  |  | Morriss, Dowrick (20) |
|  |  | Rief, Martin (21) |
|  |  | Rosendal, Olesen (22) |
|  |  | Toft, Rosendal (23) |
|  |  | Whitehead and Campion (24) |
| Behavioural therapies versus other psychological therapies for depression | Cochrane Depression, Anxiety and Neurosis Group | Rude (25) |
| Ready-to-use therapeutic food for home-based treatment of severe acute malnutrition in children from six months to five years of age | Cochrane Developmental, Psychosocial and Learning Problems Group | Ciliberto, Sandige (26) |
|  |  | Manary, Ndkeha (27) |
|  |  | Ndekha, Manary (28) |
| Specially formulated foods for treating children with moderate acute malnutrition in low- and middle-income countries | Cochrane Developmental, Psychosocial and Learning Problems Group | Ackatia-Armah, McDonald (29) |
|  |  | Delchevalerie (NTR) |
|  |  | Karakochuk, van den Briel (30) |
|  |  | Nikiema (NTR) |
| Educational and skills-based interventions for preventing relationship and dating violence in adolescents and young adults | Cochrane Developmental, Psychosocial and Learning Problems Group | Andersen (31) |
|  |  | Anderson, Stoelb (32) |
|  |  | Avery-Leaf, Cascardi (33) |
|  |  | Boulter (34) |
|  |  | Breitenbecher and Gidycz (35) |
|  |  | Fay and Medway (36) |
|  |  | Foshee, Bauman (37) |
|  |  | Foubert and Marriott (38) |
|  |  | Foubert and McEwen (39) |
|  |  | Foubert (40) |
|  |  | Gidycz, Orchowski (41) |
|  |  | Holcomb, Savage (42) |
|  |  | Jaycox, McCaffrey (43) |
|  |  | Macgowan (44) |
|  |  | Miller, Tancredi (45) |
|  |  | Pacifici, Stoolmiller (46) |
|  |  | Saberi (47) |
|  |  | Wolfe, Crooks (48) |
| Non-specialist health worker interventions for the care of mental, neurological and substance-abuse disorders in low- and middle-income countries | Cochrane Effective Practice and Organisation of Care Group | Baker-Henningham, Powell (49) |
|  |  | Berger and Gelkopf (50) |
|  |  | Bolton, Bass (51) |
|  |  | Hirani, Karmaliani (52) |
|  |  | Jenkins, Othieno (53) |
|  |  | Jordans, Komproe (54) |
|  |  | Patel, Weiss (55) |
|  |  | Rahman, Malik (56) |
|  |  | Tol, Komproe (57) |
|  |  | Tol, Komproe (58) |
| Computerized advice on drug dosage to improve prescribing practice | Cochrane Effective Practice and Organisation of Care Group | Burton, Ash (59) |
|  |  | Fitzmaurice, Hobbs (60) |
|  |  | Claes, Buntinx (61) |
|  |  | Terrell, Perkins (62) |
|  |  | Wexler, Shrader (63) |
| The effect of different methods of remuneration on the behaviour of primary care dentists | Cochrane Effective Practice and Organisation of Care Group | Clarkson, Turner (64) |
|  |  | Coventry, Holloway (65) |
| Behavioral interventions for improving condom use for dual protection | Cochrane Fertility Regulation Group | Kamali, Kinsman (66) |
|  |  | Ross, Changalucha (67) |
|  |  | Jewkes, Nduna (68) |
|  |  | Cowan, Pascoe (69) |
|  |  | Boyer, Shafer (70) |
|  |  | Stephenson, Strange (71) |
| Theory-based interventions for contraception | Cochrane Fertility Regulation Group | Coyle, Basen-Engquist (72) |
|  |  | Wight, Raab (73) |
|  |  | Stanton, Cole (74) |
|  |  | Boyer, Shafer (70) |
|  |  | Coyle, Kirby (75) |
|  |  | Ross, Changalucha (67) |
|  |  | Cowan, Pascoe (69) |
| Remote and web 2.0 interventions for promoting physical activity | Cochrane Heart Group | Elley, Kerse (76) |
| Decentralising HIV treatment in lower- and middle-income countries | Cochrane HIV/AIDS Group | Jaffar, Amuron (77) |
|  |  | Selke, Kimaiyo (78) |
| Primaquine for preventing relapse in people with *Plasmodium vivax*malaria treated with chloroquine | Cochrane Infectious Diseases Group | Leslie, Rab (79) |
|  |  | Leslie, Mayan (80) |
| Mosquito larval source management for controlling malaria | Cochrane Infectious Diseases Group | Yapabandara, Curtis (81) |
|  |  | Yapabandara and Curtis (82) |
|  |  | Shililu, Mbogo (83) |
|  |  | Coulibaly (NTR) |
| Rifamycins (rifampicin, rifabutin and rifapentine) compared to isoniazid for preventing tuberculosis in HIV-negative people at risk of active TB | Cochrane Infectious Diseases Group | Tortajada, Martinez-Lacasa (84) |
|  |  | Sterling, Villarino (85) |
| Screening for lung cancer | Cochrane Lung Cancer Group | Brett (86) |
| Targeting intensive glycaemic control versus targeting conventional glycaemic control for type 2 diabetes mellitus | Cochrane Metabolic and Endocrine Disorders Group | Griffin, Borch-Johnsen (87) |
| Non-pharmacological interventions for fatigue in rheumatoid arthritis | Cochrane Musculoskeletal Group | Häkkinen, Sokka (88) |
| Cycled light in the intensive care unit for preterm and low birth weight infants | Cochrane Neonatal Group | Brandon, Holditch-Davis (89) |
| Enamel etching for bonding fixed orthodontic braces | Cochrane Oral Health Group | Aljubouri, Millett (90) |
|  |  | Asgari, Salas (91) |
|  |  | Banks and Thiruvenkatachari (92) |
|  |  | e Cal-Neto, Quintão (93) |
|  |  | Elekdag-Turk, Isci (94) |
|  |  | Elekdag-Turk, Cakmak (95) |
|  |  | Ghiz, Ngan (96) |
|  |  | House, Ireland (97) |
|  |  | Ireland, Knight (98) |
|  |  | Manning, Chadwick (99) |
|  |  | Murfitt, Quick (100) |
|  |  | Noble, Salas-Lopez (101) |
|  |  | Paschos, Kurochkina (102) |
| Screening programmes for the early detection and prevention of oral cancer | Cochrane Oral Health Group | Sankaranarayanan, Ramadas (103) |
| Fluoride varnishes for preventing dental caries in children and adolescents | Cochrane Oral Health Group | Borutta, Reuscher (104) |
|  |  | Bravo, Baca (105) |
|  |  | Hardman, Davies (106) |
|  |  | Lawrence, Binguis (107) |
|  |  | Milsom, Blinkhorn (108) |
| Effectiveness and cost-effectiveness of home palliative care services for adults with advanced illness and their caregivers | Cochrane Pain, Palliative and Supportive Care Group | Jordhøy, Fayers (109) |
|  |  | McKegney, Bailey (110) |
|  |  | Rabow, Dibble (111) |
| Interventions for implementation of thromboprophylaxis in hospitalized medical and surgical patients at risk for venous thromboembolism | Cochrane Peripheral Vascular Diseases Group | Anderson, Wheeler (112) |
|  |  | Dexter, Perkins (113) |
|  |  | Fontaine, Mahe (114) |
|  |  | Garcia, Highfill (115) |
|  |  | Labarere, Bosson (116) |
|  |  | Overhage, Tierney (117) |
| Fetal and umbilical Doppler ultrasound in high-risk pregnancies | Cochrane Pregnancy and Childbirth Group | Giles, Bisits (118) |
|  |  | Johnstone, Prescott (119) |
|  |  | Newnham, O'Dea (120) |
| Psychosocial interventions for supporting women to stop smoking in pregnancy | Cochrane Pregnancy and Childbirth Group | Bauman, Bryan (121) |
|  |  | Campbell, Walsh (122) |
|  |  | Eades, Sanson-Fisher (123) |
|  |  | Hajek, West (124) |
|  |  | Haug, Fugelli (125) |
|  |  | Kendrick, Zahniser (126) |
|  |  | Lawrence, Aveyard (127) |
|  |  | Lillington, Royce (128) |
|  |  | Lowe, Balanda (129) |
|  |  | Manfredi, Crittenden (130) |
|  |  | McLeod, Pullon (131) |
|  |  | Messimer, Hickner (132) |
|  |  | Moore, Campbell (133) |
|  |  | Pbert, Ockene (134) |
|  |  | Polańska, Hanke (135) |
|  |  | Vilches (NTR) |
| Midwife-led continuity models versus other models of care for childbearing women | Cochrane Pregnancy and Childbirth Group | North Staffordshire Changing Childbirth Research Team (136) |
| Schedules for home visits in the early postpartum period | Cochrane Pregnancy and Childbirth Group | Christie and Bunting (137) |
|  |  | Kronborg, Væth (138) |
|  |  | MacArthur, Winter (139) |
| Telephone support for women during pregnancy and the first six weeks postpartum | Cochrane Pregnancy and Childbirth Group | Lund, Hemed (140) |
| Interventions to improve water quality and supply, sanitation and hygiene practices, and their effects on the nutritional status of children | Cochrane Public Health Group | Du Preez, McGuigan (141) |
|  |  | Du Preez, Conroy (142) |
|  |  | Luby, Agboatwalla (143) |
|  |  | Luby, Agboatwalla (144) |
|  |  | McGuigan, Samaiyar (145) |
| User-held personalised information for routine care of people with severe mental illness | Cochrane Schizophrenia Group | Warner, King (146) |
|  |  | Lester, Allan (147) |
| Tobacco cessation interventions for young people | Cochrane Tobacco Addiction Group | Aveyard, Cheng (148) |
|  |  | Chan and Witherspoon (149) |
|  |  | Hoffman, Nemes (150) |
|  |  | Horn, Dino (151) |
|  |  | Idrisov, Sun (152) |
|  |  | McCuller, Sussman (153) |
|  |  | Woodruff, Conway (154) |
| Smoking cessation interventions for smokers with current or past depression | Cochrane Tobacco Addiction Group | Batra, Collins (155) |
| Relapse prevention interventions for smoking cessation | Cochrane Tobacco Addiction Group | Conway, Woodruff (156) |
|  |  | Hajek, West (124) |
|  |  | Klesges, Haddock (157) |
|  |  | Klesges, DeBon (158) |
|  |  | Mayer, Vandecasteele (159) |
|  |  | Mermelstein, Hedeker (160) |
|  |  | Pbert, Ockene (134) |
|  |  | Razavi, Vandecasteele (161) |
|  |  | Schröter, Collins (162) |
|  |  | Severson, Andrews (163) |
| Telephone counselling for smoking cessation | Cochrane Tobacco Addiction Group | Borland, Balmford (164) |
|  |  | Chouinard and Robichaud-Ekstrand (165) |
|  |  | Ebbert, Carr (166) |
|  |  | Hennrikus, Jeffery (167) |
|  |  | Joyce, Niaura (168) |
|  |  | Katz, Muehlenbruch (169) |
|  |  | Lando, Rolnick (170) |
|  |  | Lichtenstein, Andrews (171) |
|  |  | Lichtenstein, Boles (172) |
|  |  | Ossip-Klein, Giovino (173) |
|  |  | Roski, Jeddeloh (174) |
| Nursing interventions for smoking cessation | Cochrane Tobacco Addiction Group | Bolman, De Vries (175) |
|  |  | Borrelli, Hayes (176) |
|  |  | Chouinard and Robichaud-Ekstrand (165) |
|  |  | Hilberink, Jacobs (177) |
|  |  | Steptoe, Day (178) |
|  |  | Wood, Kotseva (179) |
| Internet-based interventions for smoking cessation | Cochrane Tobacco Addiction Group | Woodruff, Conway (180) |
| Infection control strategies for preventing the transmission of meticillin-resistant *Staphylococcus aureus* (MRSA) in nursing homes for older people | Cochrane Wounds Group | Baldwin, Gilpin (181) |
| Dressings and topical agents for preventing pressure ulcers | Cochrane Wounds Group | Houwing, Van der Zwet (182) |
| Interventions for cutaneous Bowen's disease | Cochrane Skin Group | Lui, Hobbs (183) |
|  |  | Morton, Horn (184) |
|  |  | Perrett, McGregor (185) |
|  |  | Salim, Leman (186) |
| Beta-lactam versus beta-lactam-aminoglycoside combination therapy in cancer patients with neutropenia | Cochrane Gynaecological Cancer Group | Review stated there were 23 included C-RCTs, it was unclear which of the 71 included trials were classified as C-RCTs. Contacted author for more information but classification of C-RCTs was still unclear |
|  |  |  |
|  |  |  |
|  |  |  |
|  |  |  |
|  |  |  |
|  |  |  |
|  |  |  |
|  |  |  |
|  |  |  |
|  |  |  |
|  |  |  |
|  |  |  |
|  |  |  |
|  |  |  |
|  |  |  |
|  |  |  |
|  |  |  |
|  |  |  |
|  |  |  |
|  |  |  |
|  |  |  |
|  |  |  |
| Prenatal administration of progesterone for preventing preterm birth in women considered to be at risk of preterm birth | Cochrane Pregnancy and Childbirth Group | Aboulghar, Aboulghar (187) |
|  |  | Cetingoz, Cam (188) |
|  |  | Combs, Garite (189) |
|  |  | Elsheikhah, Dahab (190) |
|  |  | Fonseca, Celik (191) |
|  |  | Hartikainen-Sorri, Kauppila (192) |
|  |  | Norman, Mackenzie (193) |
|  |  | Rode, Klein (194) |
|  |  | Rouse, Caritis (195) |
|  |  | Senat, Porcher (196) |
|  |  | Serra, Perales (197) |
|  |  | Caritis, Rouse (198) |
|  |  | Combs, Garite (199) |
|  |  | Lim, Schuit (200) |
| Nutritional screening for improving professional practice for patient outcomes in hospital and primary care settings | Cochrane Pain, Palliative and Supportive Care Group | Moore, Siu (201) |

ICC=intracluster correlation coefficient; N/A=not applicable; NR=not reported; NTR=no trial report

**References**

1. Blakebrough IS, Gilles HM. The effect of rifampicin on meningococcal carriage in family contacts in northern Nigeria. The Journal of infection. 1980;2(2):137-43.

2. Cuevas LE, Kazembe P, Mughogho GK, Tillotson GS, Hart CA. Eradication of nasopharyngeal carriage of Neisseria meningitidis in children and adults in rural Africa: a comparison of ciprofloxacin and rifampicin. The Journal of infectious diseases. 1995;171(3):728-31.

3. Guttler RB, Counts GW, Avent CK, Beaty HN. Effect of rifampin and minocycline on meningococcal carrier rates. The Journal of infectious diseases. 1971;124(2):199-205.

4. Munford RS, Sussuarana de Vasconcelos ZJ, Phillips CJ, Gelli DS, Gorman GW, Risi JB, et al. Eradication of Carriage of Neisseria meningitidis in Families: A Study in Brazil. Journal of Infectious Diseases. 1974;129(6):644-9.

5. Schwartz B. Chemoprophylaxis for Bacterial Infections: Principles of and Application to Meningococcal Infections. Review of Infectious Diseases. 1991;13(Supplement 2):S170-S3.

6. Carman WF, Elder AG, Wallace LA, McAulay K, Walker A, Murray GD, et al. Effects of influenza vaccination of health-care workers on mortality of elderly people in long-term care: a randomised controlled trial. The Lancet. 2000;355(9198):93-7.

7. Potter J, Stott DJ, Roberts MA, Elder AG, O'Donnell B, Knight PV, et al. Influenza vaccination of health care workers in long-term-care hospitals reduces the mortality of elderly patients. J Infect Dis. 1997;175(1):1-6.

8. Lemaitre M, Meret T, Rothan-Tondeur M, Belmin J, Lejonc J-L, Luquel L, et al. Effect of Influenza Vaccination of Nursing Home Staff on Mortality of Residents: A Cluster-Randomized Trial. Journal of the American Geriatrics Society. 2009;57(9):1580-6.

9. Rea H, McAuley S, Stewart A, Lamont C, Roseman P, Didsbury P. A chronic disease management programme can reduce days in hospital for patients with chronic obstructive pulmonary disease. Internal Medicine Journal. 2004;34(11):608-14.

10. Wood-Baker R, McGlone S, Venn A, Walters EH. Written action plans in chronic obstructive pulmonary disease increase appropriate treatment for acute exacerbations. Respirology. 2006;11(5):619-26.

11. Bethge M, Herbold D, Trowitzsch L, Jacobi C. Work status and health-related quality of life following multimodal work hardening: A cluster randomised trial. Journal of Back and Musculoskeletal Rehabilitation. 2011;24(3):161-72.

12. Loisel P, Abenhaim L, Durand P, Esdaile JM, Suissa S, Gosselin L, et al. A Population‐Based, Randomized Clinical Trial on Back Pain Management. Spine. 1997;22(24):2911-8.

13. Segnan N, Armaroli P, Bonelli L, Risio M, Sciallero S, Zappa M, et al. Once-Only Sigmoidoscopy in Colorectal Cancer Screening: Follow-up Findings of the Italian Randomized Controlled Trial—SCORE. Journal of the National Cancer Institute. 2011;103(17):1310-22.

14. Coleman TE. The Effects of Age and Exposure to Mental Illness Educational Material on Student Nurses' Attitudes Toward the Mentally Ill: Hofstra University; 2005.

15. Penn DL, Chamberlin C, Mueser KT. Effects of a documentary film about schizophrenia on psychiatric stigma. Schizophrenia bulletin. 2003;29(2):383.

16. Solberg LI, Asche SA, Sepucha K, Thygeson NM, Madden JE, Morrissey L, et al. Informed choice assistance for women making uterine fibroid treatment decisions: a practical clinical trial. Medical Decision Making. 2009.

17. Paci E, Barneschi M, Miccinesi G, Falchi S, Metrangolo L, Novelli G. Informed consent and patient participation in the medical encounter: a list of questions for an informed choice about the type of anaesthesia. European journal of anaesthesiology. 1999;16(3):160-5.

18. Blankenstein AH. Somatising patients in general practice: reattribution, a promising approach. 2001.

19. Larisch A, Schweickhardt A, Wirsching M, Fritzsche K. Psychosocial interventions for somatizing patients by the general practitioner: a randomized controlled trial. Journal of psychosomatic research. 2004;57(6):507-14.

20. Morriss R, Dowrick C, Salmon P, Peters S, Dunn G, Rogers A, et al. Cluster randomised controlled trial of training practices in reattribution for medically unexplained symptoms. The British Journal of Psychiatry. 2007;191(6):536-42.

21. Rief W, Martin A, Rauh E, Zech T, Bender A. Evaluation of general practitioners’ training: how to manage patients with unexplained physical symptoms. Psychosomatics. 2006;47(4):304-11.

22. Rosendal M, Olesen F, Fink P, Toft T, Sokolowski I, Bro F. A randomized controlled trial of brief training in the assessment and treatment of somatization in primary care: effects on patient outcome. General hospital psychiatry. 2007;29(4):364-73.

23. Toft T, Rosendal M, Ørnbøl E, Olesen F, Frostholm L, Fink P. Training general practitioners in the treatment of functional somatic symptoms: effects on patient health in a cluster-randomised controlled trial (the Functional Illness in Primary Care study). Psychotherapy and psychosomatics. 2010;79(4):227-37.

24. Whitehead L, Campion P. Can general practitioners manage chronic fatigue syndrome? A controlled trial. Journal of Chronic Fatigue Syndrome. 2002;10(1):55-64.

25. Rude SS. Relative benefits of assertion or cognitive self-control treatment for depression as a function of proficiency in each domain. Journal of consulting and clinical psychology. 1986;54(3):390.

26. Ciliberto MA, Sandige H, Ndekha MJ, Ashorn P, Briend A, Ciliberto HM, et al. Comparison of home-based therapy with ready-to-use therapeutic food with standard therapy in the treatment of malnourished Malawian children: a controlled, clinical effectiveness trial. The American journal of clinical nutrition. 2005;81(4):864-70.

27. Manary MJ, Ndkeha M, Ashorn P, Maleta K, Briend A. Home based therapy for severe malnutrition with ready-to-use food. Archives of Disease in Childhood. 2004;89(6):557-61.

28. Ndekha M, Manary M, Ashorn P, Briend A. Home‐based therapy with ready‐to‐use therapeutic food is of benefit to malnourished, HIV‐infected Malawian children. Acta Paediatrica. 2005;94(2):222-5.

29. Ackatia-Armah RS, McDonald C, Doumbia S, Brown KH. Effect of selected dietary regimens on recovery from moderate acute malnutrition in young Malian children. The FASEB Journal. 2012;26(1_MeetingAbstracts):1031.10.

30. Karakochuk C, van den Briel T, Stephens D, Zlotkin S. Treatment of moderate acute malnutrition with ready-to-use supplementary food results in higher overall recovery rates compared with a corn-soya blend in children in southern Ethiopia: an operations research trial. The American journal of clinical nutrition. 2012;96(4):911-6.

31. Andersen SJ. The effects of acquaintance rape prevention programming on male athletes' sexual and dating attitudes: University of Wisconsin-La Crosse; 1992.

32. Anderson L, Stoelb MP, Duggan P, Hieger B, Kling KH, Payne JP. The effectiveness of two types of rape prevention programs in changing the rape-supportive attitudes of college students. Journal of College Student Development. 1998.

33. Avery-Leaf S, Cascardi M, O'leary KD, Cano A. Efficacy of a dating violence prevention program on attitudes justifying aggression. Journal of Adolescent Health. 1997;21(1):11-7.

34. Boulter C. Effects of an acquaintance rape prevention program on male college students' endorsements of rape myth beliefs and sexually coercive behaviors: Washington State University; 1997.

35. Breitenbecher KH, Gidycz CA. An empirical evaluation of a program designed to reduce the risk of multiple sexual victimization. Journal of Interpersonal Violence. 1998;13(4):472-88.

36. Fay KE, Medway FJ. An acquaintance rape education program for students transitioning to high school. Sex education. 2006;6(3):223-36.

37. Foshee VA, Bauman KE, Arriaga XB, Helms RW, Koch GG, Linder GF. An evaluation of Safe Dates, an adolescent dating violence prevention program. American journal of public health. 1998;88(1):45-50.

38. Foubert JD, Marriott KA. Effects of a sexual assault peer education program on men’s belief in rape myths. Sex Roles. 1997;36(3-4):259-68.

39. Foubert JD, McEwen MK. An all-male rape-prevention peer education program: Decreasing fraternity men’s behavioral intent to rape. Journal of College Student Development. 1998;39:548-56.

40. Foubert JD. The longitudinal effects of a rape-prevention program on fraternity men's attitudes, behavioral intent, and behavior. Journal of American College Health. 2000;48(4):158-63.

41. Gidycz CA, Orchowski LM, Berkowitz AD. Preventing sexual aggression among college men: An evaluation of a social norms and bystander intervention program. Violence Against Women. 2011:1077801211409727.

42. Holcomb DR, Savage MP, Seehafer R, Waalkes DM. A mixed-gender date rape prevention intervention targeting freshmen college athletes. College Student Journal. 2002;36(2):165.

43. Jaycox LH, McCaffrey D, Eiseman B, Aronoff J, Shelley GA, Collins RL, et al. Impact of a school-based dating violence prevention program among Latino teens: Randomized controlled effectiveness trial. Journal of Adolescent Health. 2006;39(5):694-704.

44. Macgowan MJ. An evaluation of a dating violence prevention program for middle school students. Violence and victims. 1997;12(3):223-35.

45. Miller E, Tancredi DJ, McCauley HL, Decker MR, Virata MCD, Anderson HA, et al. “Coaching boys into men”: a cluster-randomized controlled trial of a dating violence prevention program. Journal of Adolescent Health. 2012;51(5):431-8.

46. Pacifici C, Stoolmiller M, Nelson C. Evaluating a prevention program for teenagers on sexual coercion: a differential effectiveness approach. Journal of consulting and clinical psychology. 2001;69(3):552.

47. Saberi D. Acquaintance rape prevention: Changing rape-supportive attitudes of college students: Arizona State University; 1999.

48. Wolfe DA, Crooks C, Jaffe P, Chiodo D, Hughes R, Ellis W, et al. A school-based program to prevent adolescent dating violence: A cluster randomized trial. Archives of Pediatrics & Adolescent Medicine. 2009;163(8):692-9.

49. Baker-Henningham H, Powell C, Walker S, Grantham-McGregor S. The effect of early stimulation on maternal depression: a cluster randomised controlled trial. Archives of Disease in Childhood. 2005;90(12):1230-4.

50. Berger R, Gelkopf M. School-based intervention for the treatment of tsunami-related distress in children: a quasi-randomized controlled trial. Psychotherapy and psychosomatics. 2009.

51. Bolton P, Bass J, Neugebauer R, Verdeli H, Clougherty KF, Wickramaratne P, et al. Group interpersonal psychotherapy for depression in rural Uganda: a randomized controlled trial. Jama. 2003;289(23):3117-24.

52. Hirani SS, Karmaliani R, McFarlane J, Asad N, Madhani F. testing a community derived intervention to promote women’s health: preliminary results of a 3-arm randomized controlled trial in Karachi, Pakistan. Pakistan; 2010.

53. Jenkins R, Othieno C, Okeyo S, Kaseje D, Aruwa J, Oyugi H, et al. Short structured general mental health in service training programme in Kenya improves patient health and social outcomes but not detection of mental health problems-a pragmatic cluster randomised controlled trial. International journal of mental health systems. 2013;7(1):25.

54. Jordans MJ, Komproe IH, Tol WA, Kohrt BA, Luitel NP, Macy RD, et al. Evaluation of a classroom‐based psychosocial intervention in conflict‐affected Nepal: a cluster randomized controlled trial. Journal of Child Psychology and Psychiatry. 2010;51(7):818-26.

55. Patel V, Weiss HA, Chowdhary N, Naik S, Pednekar S, Chatterjee S, et al. Effectiveness of an intervention led by lay health counsellors for depressive and anxiety disorders in primary care in Goa, India (MANAS): a cluster randomised controlled trial. The Lancet. 2010;376(9758):2086-95.

56. Rahman A, Malik A, Sikander S, Roberts C, Creed F. Cognitive behaviour therapy-based intervention by community health workers for mothers with depression and their infants in rural Pakistan: a cluster-randomised controlled trial. The Lancet. 2008;372(9642):902-9.

57. Tol WA, Komproe IH, Susanty D, Jordans MJ, Macy RD, De Jong JT. School-based mental health intervention for children affected by political violence in Indonesia: a cluster randomized trial. Jama. 2008;300(6):655-62.

58. Tol WA, Komproe IH, Jordans MJ, Vallipuram A, Sipsma H, Sivayokan S, et al. Outcomes and moderators of a preventive school‐based mental health intervention for children affected by war in Sri Lanka: a cluster randomized trial. World Psychiatry. 2012;11(2):114-22.

59. Burton ME, Ash CL, Hill DP, Handy T, Shepherd MD, Vasko MR. A controlled trial of the cost benefit of computerized bayesian aminoglycoside administration. Clinical Pharmacology & Therapeutics. 1991;49(6):685-94.

60. Fitzmaurice DA, Hobbs FR, Murray ET, Holder RL, Allan TF, Rose PE. Oral anticoagulation management in primary care with the use of computerized decision support and near-patient testing: a randomized, controlled trial. Archives of Internal Medicine. 2000;160(15):2343-8.

61. Claes N, Buntinx F, Vijgen J, Arnout J, Vermylen J, Fieuws S, et al. The Belgian improvement study on oral anticoagulation therapy: a randomized clinical trial. European heart journal. 2005;26(20):2159-65.

62. Terrell KM, Perkins AJ, Hui SL, Callahan CM, Dexter PR, Miller DK. Computerized decision support for medication dosing in renal insufficiency: a randomized, controlled trial. Annals of emergency medicine. 2010;56(6):623-9. e2.

63. Wexler DJ, Shrader P, Burns SM, Cagliero E. Effectiveness of a Computerized Insulin Order Template in General Medical Inpatients With Type 2 Diabetes A cluster randomized trial. Diabetes Care. 2010;33(10):2181-3.

64. Clarkson J, Turner S, Grimshaw J, Ramsay C, Johnston M, Scott A, et al. Changing clinicians’ behavior: a randomized controlled trial of fees and education. Journal of Dental Research. 2008;87(7):640-4.

65. Coventry P, Holloway P, Lennon M, Mellor A, Worthington H. A trial of a capitation system of payment for the treatment of children in the General Dental Service. Final report. Dental Health Services Research Unit, University of Manchester. September, 1989. Community dental health. 1989;6:1-63.

66. Kamali A, Kinsman J, Nalweyiso N, Mitchell K, Kanyesigye E, Kengeya‐Kayondo JF, et al. A community randomized controlled trial to investigate impact of improved STD management and behavioural interventions on HIV incidence in rural Masaka, Uganda: trial design, methods and baseline findings. Tropical Medicine & International Health. 2002;7(12):1053-63.

67. Ross DA, Changalucha J, Obasi AI, Todd J, Plummer ML, Cleophas-Mazige B, et al. Biological and behavioural impact of an adolescent sexual health intervention in Tanzania: a community-randomized trial. Aids. 2007;21(14):1943-55.

68. Jewkes R, Nduna M, Levin J, Jama N, Dunkle K, Puren A, et al. Impact of stepping stones on incidence of HIV and HSV-2 and sexual behaviour in rural South Africa: cluster randomised controlled trial. Bmj. 2008;337:a506.

69. Cowan FM, Pascoe SJ, Langhaug LF, Mavhu W, Chidiya S, Jaffar S, et al. The Regai Dzive Shiri Project: results of a randomised trial of an HIV prevention intervention for Zimbabwean youth. AIDS (London, England). 2010;24(16):2541.

70. Boyer CB, Shafer M-A, Shaffer RA, Brodine SK, Pollack LM, Betsinger K, et al. Evaluation of a cognitive–behavioral, group, randomized controlled intervention trial to prevent sexually transmitted infections and unintended pregnancies in young women. Preventive medicine. 2005;40(4):420-31.

71. Stephenson J, Strange V, Allen E, Copas A, Johnson A, Bonell C, et al. The long-term effects of a peer-led sex education programme (RIPPLE): a cluster randomised trial in schools in England. PLoS medicine. 2008;5(11):e224.

72. Coyle K, Basen-Engquist K, Kirby D, Parcel G, Banspach S, Collins J, et al. Safer choices: reducing teen pregnancy, HIV, and STDs. Public health reports. 2001;116(Suppl 1):82.

73. Wight D, Raab GM, Henderson M, Abraham C, Buston K, Hart G, et al. Limits of teacher delivered sex education: interim behavioural outcomes from randomised trial. Bmj. 2002;324(7351):1430.

74. Stanton B, Cole M, Galbraith J, Li X, Pendleton S, Cottrel L, et al. Randomized trial of a parent intervention: parents can make a difference in long-term adolescent risk behaviors, perceptions, and knowledge. Archives of pediatrics & adolescent medicine. 2004;158(10):947-55.

75. Coyle KK, Kirby DB, Robin LE, Banspach SW, Baumler E, Glassman JR. All4You! A randomized trial of an HIV, other STDs, and pregnancy prevention intervention for alternative school students. AIDS Education & Prevention. 2006;18(3):187-203.

76. Elley CR, Kerse N, Arroll B, Robinson E. Effectiveness of counselling patients on physical activity in general practice: cluster randomised controlled trial. Bmj. 2003;326(7393):793.

77. Jaffar S, Amuron B, Foster S, Birungi J, Levin J, Namara G, et al. Rates of virological failure in patients treated in a home-based versus a facility-based HIV-care model in Jinja, southeast Uganda: a cluster-randomised equivalence trial. The Lancet. 2010;374(9707):2080-9.

78. Selke HM, Kimaiyo S, Sidle JE, Vedanthan R, Tierney WM, Shen C, et al. Task-shifting of antiretroviral delivery from health care workers to persons living with HIV/AIDS: clinical outcomes of a community-based program in Kenya. JAIDS Journal of Acquired Immune Deficiency Syndromes. 2010;55(4):483-90.

79. Leslie T, Rab MA, Ahmadzai H, Durrani N, Fayaz M, Kolaczinski J, et al. Compliance with 14-day primaquine therapy for radical cure of vivax malaria—a randomized placebo-controlled trial comparing unsupervised with supervised treatment. Transactions of the Royal Society of Tropical Medicine and Hygiene. 2004;98(3):168-73.

80. Leslie T, Mayan I, Mohammed N, Erasmus P, Kolaczinski J, Whitty CJ, et al. A randomised trial of an eight-week, once weekly primaquine regimen to prevent relapse of Plasmodium vivax in Northwest Frontier Province, Pakistan. PLoS One. 2008;3(8):e2861.

81. Yapabandara A, Curtis C, Wickramasinghe M, Fernando W. Control of malaria vectors with the insect growth regulator pyriproxyfen in a gem-mining area in Sri Lanka. Acta tropica. 2001;80(3):265-76.

82. Yapabandara A, Curtis C. Control of Vectors and Incidence of Malaria in an Irrigated Settlement Scheme in Sri Lanka When Using the Insect Growth Regulator Pyriproxyfen. Journal of the American Mosquito Control Association. 2004;20(4):395-400.

83. Shililu J, Mbogo C, Ghebremeskel T, Githure J, Novak R. Mosquito larval habitats in a semiarid ecosystem in Eritrea: impact of larval habitat management on Anopheles arabiensis population. The American journal of tropical medicine and hygiene. 2007;76(1):103-10.

84. Tortajada C, Martinez-Lacasa J, Sanchez F, Jimenez-Fuentes A, De Souza M, García J, et al. Is the combination of pyrazinamide plus rifampicin safe for treating latent tuberculosis infection in persons not infected by the human immunodeficiency virus? The International Journal of Tuberculosis and Lung Disease. 2005;9(3):276-81.

85. Sterling TR, Villarino ME, Borisov AS, Shang N, Gordin F, Bliven-Sizemore E, et al. Three months of rifapentine and isoniazid for latent tuberculosis infection. New England Journal of Medicine. 2011;365(23):2155-66.

86. Brett G. The value of lung cancer detection by six-monthly chest radiographs. Thorax. 1968;23(4):414-20.

87. Griffin SJ, Borch-Johnsen K, Davies MJ, Khunti K, Rutten GE, Sandbæk A, et al. Effect of early intensive multifactorial therapy on 5-year cardiovascular outcomes in individuals with type 2 diabetes detected by screening (ADDITION-Europe): a cluster-randomised trial. The Lancet. 2011;378(9786):156-67.

88. Häkkinen A, Sokka T, Lietsalmi AM, Kautiainen H, Hannonen P. Effects of dynamic strength training on physical function, Valpar 9 work sample test, and working capacity in patients with recent‐onset rheumatoid arthritis. Arthritis Care & Research. 2003;49(1):71-7.

89. Brandon DH, Holditch-Davis D, Belyea M. Preterm infants born at less than 31 weeks' gestation have improved growth in cycled light compared with continuous near darkness. The Journal of pediatrics. 2002;140(2):192-9.

90. Aljubouri Y, Millett D, Gilmour W. Six and 12 months' evaluation of a self-etching primer versus two-stage etch and prime for orthodontic bonding: a randomized clinical trial. The European Journal of Orthodontics. 2004;26(6):565-71.

91. Asgari S, Salas A, English J, Powers J. Clinical evaluation of bond failure rates with a new self-etching primer. Journal of clinical orthodontics: JCO. 2002;36(12):687.

92. Banks P, Thiruvenkatachari B. Long-term clinical evaluation of bracket failure with a self-etching primer: a randomized controlled trial. Journal of orthodontics. 2007;34(4):243-51.

93. e Cal-Neto JP, Quintão CA, de Oliveira Almeida MA, Miguel JAM. Bond failure rates with a self-etching primer: a randomized controlled trial. American Journal of Orthodontics and Dentofacial Orthopedics. 2009;135(6):782-6.

94. Elekdag-Turk S, Isci D, Turk T, Cakmak F. Six-month bracket failure rate evaluation of a self-etching primer. The European Journal of Orthodontics. 2008;30(2):211-6.

95. Elekdag-Turk S, Cakmak F, Isci D, Turk T. 12-month self-ligating bracket failure rate with a self-etching primer. The Angle orthodontist. 2008;78(6):1095-100.

96. Ghiz MA, Ngan P, Kao E, Martin C, Gunel E. Effects of sealant and self-etching primer on enamel decalcification. Part II: an in-vivo study. American Journal of Orthodontics and Dentofacial Orthopedics. 2009;135(2):206-13.

97. House K, Ireland A, Sherriff M. An investigation into the use of a single component self-etching primer adhesive system for orthodontic bonding: a randomized controlled clinical trial. Journal of orthodontics. 2006;33(1):38-44.

98. Ireland AJ, Knight H, Sherriff M. An in vivo investigation into bond failure rates with a new self-etching primer system. American Journal of Orthodontics and Dentofacial Orthopedics. 2003;124(3):323-6.

99. Manning N, Chadwick S, Plunkett D, Macfarlane T. A randomized clinical trial comparing ‘one-step’and ‘two-step’orthodontic bonding systems. Journal of Orthodontics. 2006;33(4):276-83.

100. Murfitt P, Quick A, Swain M, Herbison G. A randomised clinical trial to investigate bond failure rates using a self-etching primer. The European Journal of Orthodontics. 2006;28(5):444-9.

101. Noble RR, Salas-Lopez A, English JD, Powers JM. Clinical evaluation of orthodontic self-etching primers. Texas dental journal. 2006;123(3):274-8.

102. Paschos E, Kurochkina N, Huth KC, Hansson CS, Rudzki-Janson I. Failure rate of brackets bonded with antimicrobial and fluoride-releasing, self-etching primer and the effect on prevention of enamel demineralization. American Journal of Orthodontics and Dentofacial Orthopedics. 2009;135(5):613-20.

103. Sankaranarayanan R, Ramadas K, Thomas G, Muwonge R, Thara S, Mathew B, et al. Effect of screening on oral cancer mortality in Kerala, India: a cluster-randomised controlled trial. The Lancet. 2005;365(9475):1927-33.

104. Borutta A, Reuscher G, Hufnagl S, Möbius S. [Caries prevention with fluoride varnishes among preschool children]. Gesundheitswesen (Bundesverband der Arzte des Offentlichen Gesundheitsdienstes (Germany)). 2006;68(11):731-4.

105. Bravo M, Baca P, Llodra JC, Osorio E. A 24‐month Study Comparing Sealant and Fluoride Varnish in Caries Reduction on Different Permanent First Molar Surfaces. Journal of public health dentistry. 1997;57(3):184-6.

106. Hardman M, Davies G, Duxbury J, Davies R. A cluster randomised controlled trial to evaluate the effectiveness of fluoride varnish as a public health measure to reduce caries in children. Caries research. 2006;41(5):371-6.

107. Lawrence HP, Binguis D, Douglas J, McKeown L, Switzer B, Figueiredo R, et al. A 2‐year community‐randomized controlled trial of fluoride varnish to prevent early childhood caries in Aboriginal children. Community dentistry and oral epidemiology. 2008;36(6):503-16.

108. Milsom K, Blinkhorn A, Walsh T, Worthington H, Kearney-Mitchell P, Whitehead H, et al. A Cluster-randomized Controlled Trial Fluoride Varnish in School Children. Journal of dental research. 2011;90(11):1306-11.

109. Jordhøy MS, Fayers P, Saltnes T, Ahlner-Elmqvist M, Jannert M, Kaasa S. A palliative-care intervention and death at home: a cluster randomised trial. The Lancet. 2000;356(9233):888-93.

110. McKegney FP, Bailey LR, Yates JW. Prediction and management of pain in patients with advanced cancer. General hospital psychiatry. 1981;3(2):95-101.

111. Rabow MW, Dibble SL, Pantilat SZ, McPhee SJ. The comprehensive care team: a controlled trial of outpatient palliative medicine consultation. Archives of internal medicine. 2004;164(1):83-91.

112. Anderson FA, Wheeler HB, Goldberg RJ, Hosmer DW, Forcier A, Patwardhan NA. Changing clinical practice: prospective study of the impact of continuing medical education and quality assurance programs on use of prophylaxis for venous thromboembolism. Archives of internal medicine. 1994;154(6):669-77.

113. Dexter PR, Perkins S, Overhage JM, Maharry K, Kohler RB, McDonald CJ. A computerized reminder system to increase the use of preventive care for hospitalized patients. New England Journal of Medicine. 2001;345(13):965-70.

114. Fontaine A, Mahe I, Bergmann J, Fiessinger J, Dhote R, Cohen P, et al. Effectiveness of written guidelines on the appropriateness of thromboprophylaxis prescriptions for medical patients: a prospective randomized study. Journal of internal medicine. 2006;260(4):369-76.

115. Garcia DA, Highfill J, Finnerty K, Varoz E, McConkey S, Hutchinson K, et al. A prospective, controlled trial of a pharmacy-driven alert system to increase thromboprophylaxis rates in medical inpatients. Blood Coagulation & Fibrinolysis. 2009;20(7):541-5.

116. Labarere J, Bosson J-L, Sevestre M-A, Sellier E, Richaud C, Legagneux A. Intervention targeted at nurses to improve venous thromboprophylaxis. International Journal for Quality in Health Care. 2007;19(5):301-8.

117. Overhage JM, Tierney WM, McDonald CJ. Computer reminders to implement preventive care guidelines for hospitalized patients. Archives of Internal Medicine. 1996;156(14):1551-6.

118. Giles W, Bisits A, O'Callaghan S, Gill A. The Doppler assessment in multiple pregnancy randomised controlled trial of ultrasound biometry versus umbilical artery Doppler ultrasound and biometry in twin pregnancy. BJOG: An International Journal of Obstetrics & Gynaecology. 2003;110(6):593-7.

119. Johnstone FD, Prescott R, Hoskins P, Greer IA, McGlew T, Compton M. The effect of introduction of umbilical Doppler recordings to obstetric practice. BJOG: An International Journal of Obstetrics & Gynaecology. 1993;100(8):733-41.

120. Newnham JP, O'Dea MRA, Reid KP, Diepeveen DA. Doppler flow velocity waveform analysis in high risk pregnancies: a randomized controlled trial. BJOG: An International Journal of Obstetrics & Gynaecology. 1991;98(10):956-63.

121. Bauman KE, Bryan ES, Dent CW, Koch GG. The influence of observing carbon monoxide level on cigarette smoking by public prenatal patients. American journal of public health. 1983;73(9):1089-91.

122. Campbell E, Walsh R, Sanson-Fisher R, Burrows S, Stojanovski E. A group randomised trial of two methods for disseminating a smoking cessation programme to public antenatal clinics: effects on patient outcomes. Tobacco control. 2006;15(2):97-102.

123. Eades SJ, Sanson-Fisher RW, Wenitong M, Panaretto K, D'Este C, Gilligan C, et al. An intensive smoking intervention for pregnant Aboriginal and Torres Strait Islander women: a randomised controlled trial. Medical Journal of Australia. 2012;197(1):42.

124. Hajek P, West R, Lee A, Foulds J, Owen L, Eiser JR, et al. Randomized controlled trial of a midwife‐delivered brief smoking cessation intervention in pregnancy. Addiction. 2001;96(3):485-94.

125. Haug K, Fugelli P, Aarø LE, Foss OP. Is smoking intervention in general practice more successful among pregnant than non-pregnant women? Family Practice. 1994;11(2):111-6.

126. Kendrick JS, Zahniser SC, Miller N, Salas N, Stine J, Gargiullo PM, et al. Integrating smoking cessation into routine public prenatal care: the Smoking Cessation in Pregnancy project. American Journal of Public Health. 1995;85(2):217-22.

127. Lawrence T, Aveyard P, Evans O, Cheng K. A cluster randomised controlled trial of smoking cessation in pregnant women comparing interventions based on the transtheoretical (stages of change) model to standard care. Tobacco Control. 2003;12(2):168-77.

128. Lillington L, Royce J, Novak D, Ruvalcaba M, Chlebowski R. Evaluation of a smoking cessation program for pregnant minority women. Cancer practice. 1994;3(3):157-63.

129. Lowe JB, Balanda KP, Stanton WR, Del Mar C, O’Connor V. Dissemination of an efficacious antenatal smoking cessation program in public hospitals in Australia: a randomized controlled trial. Health education & behavior. 2002;29(5):608-19.

130. Manfredi C, Crittenden KS, Warnecke R, Engler J, Cho YI, Shaligram C. Evaluation of a motivational smoking cessation intervention for women in public health clinics. Preventive Medicine. 1999;28(1):51-60.

131. McLeod D, Pullon S, Benn C, Cookson T, Dowell A, Viccars A, et al. Can support and education for smoking cessation and reduction be provided effectively by midwives within primary maternity care? Midwifery. 2004;20(1):37-50.

132. Messimer S, Hickner J, Henry R. A comparison of two antismoking interventions among pregnant women in eleven private primary care practices. The Journal of family practice. 1989;28(3):283-8.

133. Moore L, Campbell R, Whelan A, Mills N, Lupton P, Misselbrook E, et al. Self help smoking cessation in pregnancy: cluster randomised controlled trial. Bmj. 2002;325(7377):1383.

134. Pbert L, Ockene JK, Zapka J, Ma Y, Goins KV, Oncken C, et al. A community health center smoking-cessation intervention for pregnant and postpartum women. American journal of preventive medicine. 2004;26(5):377-85.

135. Polańska K, Hanke W, Sobala W, Lowe J. Efficacy and effectiveness of the smoking cessation program for pregnant women. Int J Occup Med Environ Health. 2004;17(3):369-77.

136. North Staffordshire Changing Childbirth Research Team. A randomised study of midwifery caseload care and traditional'shared-care'. Midwifery. 2000;16(4):295.

137. Christie J, Bunting B. The effect of health visitors’ postpartum home visit frequency on first-time mothers: Cluster randomised trial. International Journal of Nursing Studies. 2011;48(6):689-702.

138. Kronborg H, Væth M, Olsen J, Iversen L, Harder I. Effect of early postnatal breastfeeding support: a cluster‐randomized community based trial. Acta Paediatrica. 2007;96(7):1064-70.

139. MacArthur C, Winter H, Bick D, Knowles H, Lilford R, Henderson C, et al. Effects of redesigned community postnatal care on womens' health 4 months after birth: a cluster randomised controlled trial. The lancet. 2002;359(9304):378-85.

140. Lund S, Hemed M, Nielsen BB, Said A, Said K, Makungu M, et al. Mobile phones as a health communication tool to improve skilled attendance at delivery in Zanzibar: a cluster‐randomised controlled trial. BJOG: An International Journal of Obstetrics & Gynaecology. 2012;119(10):1256-64.

141. Du Preez M, McGuigan KG, Conroy RM. Solar disinfection of drinking water in the prevention of dysentery in South African children aged under 5 years: the role of participant motivation. Environmental science & technology. 2010;44(22):8744-9.

142. Du Preez M, Conroy RM, Ligondo S, Hennessy J, Elmore-Meegan M, Soita A, et al. Randomized intervention study of solar disinfection of drinking water in the prevention of dysentery in Kenyan children aged under 5 years. Environmental science & technology. 2011;45(21):9315-23.

143. Luby SP, Agboatwalla M, Painter J, Altaf A, Billhimer WL, Hoekstra RM. Effect of intensive handwashing promotion on childhood diarrhea in high-risk communities in Pakistan: a randomized controlled trial. Jama. 2004;291(21):2547-54.

144. Luby SP, Agboatwalla M, Painter J, Altaf A, Billhimer W, Keswick B, et al. Combining drinking water treatment and hand washing for diarrhoea prevention, a cluster randomised controlled trial. Tropical Medicine & International Health. 2006;11(4):479-89.

145. McGuigan KG, Samaiyar P, du Preez M, Conroy RM. High compliance randomized controlled field trial of solar disinfection of drinking water and its impact on childhood diarrhea in rural Cambodia. Environmental science & technology. 2011;45(18):7862-7.

146. Warner JP, King M, Blizard R, McClenahan Z, Tang S. Patient-held shared care records for individuals with mental illness Randomised controlled evaluation. The British Journal of Psychiatry. 2000;177(4):319-24.

147. Lester H, Allan T, Wilson S, Jowett S, Roberts L. A cluster randomised controlled trial of patient-held medical records for people with schizophrenia receiving shared care. British Journal of General Practice. 2003;53(488):197-203.

148. Aveyard P, Cheng K, Almond J, Sherratt E, Lancashire R, Lawrence T, et al. Cluster randomised controlled trial of expert system based on the transtheoretical (“stages of change”) model for smoking prevention and cessation in schools. Bmj. 1999;319(7215):948-53.

149. Chan C-W, Witherspoon JM. Health risk appraisal modifies cigarette smoking behavior among college students. Journal of general internal medicine. 1988;3(6):555-9.

150. Hoffman J, Nemes S, Weil J, Zack S, Munly K, Hess L. Evaluation of the ASCENT smoking cessation program for adolescents. Journal of Smoking Cessation. 2008;3(01):2-8.

151. Horn K, Dino G, Branstetter SA, Zhang J, Noerachmanto N, Jarrett T, et al. Effects of physical activity on teen smoking cessation. Pediatrics. 2011;128(4):e801-e11.

152. Idrisov B, Sun P, Akhmadeeva L, Arpawong TE, Kukhareva P, Sussman S. Immediate and six-month effects of Project EX Russia: A smoking cessation intervention pilot program. Addictive behaviors. 2013;38(8):2402-8.

153. McCuller WJ, Sussman S, Wapner M, Dent C, Weiss DJ. Motivation to quit as a mediator of tobacco cessation among at-risk youth. Addictive Behaviors. 2006;31(5):880-8.

154. Woodruff SI, Conway TL, Edwards CC. Sociodemographic and smoking-related psychosocial predictors of smoking behavior change among high school smokers. Addictive behaviors. 2008;33(2):354-8.

155. Batra A, Collins SE, Schröter M, Eck S, Torchalla I, Buchkremer G. A cluster-randomized effectiveness trial of smoking cessation modified for at-risk smoker subgroups. Journal of substance abuse treatment. 2010;38(2):128-40.

156. Conway TL, Woodruff SI, Edwards CC, Elder JP, Hurtado SL, Hervig LK. Operation Stay Quit: evaluation of two smoking relapse prevention strategies for women after involuntary cessation during US Navy recruit training. Military medicine. 2004;169(3):236-42.

157. Klesges RC, Haddock CK, Lando H, Talcott GW. Efficacy of forced smoking cessation and an adjunctive behavioral treatment on long-term smoking rates. Journal of consulting and clinical psychology. 1999;67(6):952.

158. Klesges RC, DeBon M, Vander Weg MW, Haddock CK, Lando HA, Relyea GE, et al. Efficacy of a tailored tobacco control program on long-term use in a population of US military troops. Journal of consulting and clinical psychology. 2006;74(2):295.

159. Mayer C, Vandecasteele H, Bodo M, Primo C, Slachmuylder J-L, Kaufman L, et al. Smoking relapse prevention programs and factors that predict abstinence: a controlled study comparing the efficacy of workplace group counselling and proactive phone counselling. Journal of Smoking Cessation. 2010;5(01):83-94.

160. Mermelstein R, Hedeker D, Wong SC. Extended telephone counseling for smoking cessation: does content matter? Journal of consulting and clinical psychology. 2003;71(3):565.

161. Razavi D, Vandecasteele H, Primo C, Bodo M, Debrier F, Verbist H, et al. Maintaining abstinence from cigarette smoking: effectiveness of group counselling and factors predicting outcome. European Journal of Cancer. 1999;35(8):1238-47.

162. Schröter M, Collins SE, Frittrang T, Buchkremer G, Batra A. Randomized controlled trial of relapse prevention and a standard behavioral intervention with adult smokers. Addictive behaviors. 2006;31(7):1259-64.

163. Severson HH, Andrews JA, Lichtenstein E, Wall M, Akers L. Reducing maternal smoking and relapse: long-term evaluation of a pediatric intervention. Preventive medicine. 1997;26(1):120-30.

164. Borland R, Balmford J, Bishop N, Segan C, Piterman L, McKay-Brown L, et al. In-practice management versus quitline referral for enhancing smoking cessation in general practice: a cluster randomized trial. Family practice. 2008;25(5):382-9.

165. Chouinard M-C, Robichaud-Ekstrand S. The effectiveness of a nursing inpatient smoking cessation program in individuals with cardiovascular disease. Nursing research. 2005;54(4):243-54.

166. Ebbert JO, Carr AB, Patten CA, Morris RA, Schroeder DR. Tobacco use quitline enrollment through dental practices: a pilot study. The Journal of the American Dental Association. 2007;138(5):595-601.

167. Hennrikus DJ, Jeffery RW, Lando HA, Murray DM, Brelje K, Davidann B, et al. The SUCCESS project: the effect of program format and incentives on participation and cessation in worksite smoking cessation programs. American Journal of Public Health. 2002;92(2):274-9.

168. Joyce GF, Niaura R, Maglione M, Mongoven J, Larson‐Rotter C, Coan J, et al. The effectiveness of covering smoking cessation services for Medicare beneficiaries. Health services research. 2008;43(6):2106-23.

169. Katz DA, Muehlenbruch DR, Brown RL, Fiore MC, Baker TB. Effectiveness of implementing the agency for healthcare research and quality smoking cessation clinical practice guideline: a randomized, controlled trial. Journal of the National Cancer Institute. 2004;96(8):594-603.

170. Lando HA, Rolnick S, Klevan D, Roski J, Cherney L, Lauger G. Telephone support as an adjunct to transdermal nicotine in smoking cessation. American Journal of Public Health. 1997;87(10):1670-4.

171. Lichtenstein E, Andrews JA, Lee ME, Glasgow RE, Hampson SE. Using radon risk to motivate smoking reduction: evaluation of written materials and brief telephone counselling. Tobacco control. 2000;9(3):320-6.

172. Lichtenstein E, Boles SM, Lee ME, Hampson SE, Glasgow RE, Fellows J. Using radon risk to motivate smoking reduction II: randomized evaluation of brief telephone counseling and a targeted video. Health education research. 2008;23(2):191-201.

173. Ossip-Klein DJ, Giovino GA, Megahed N, Black PM, Emont SL, Stiggins J, et al. Effects of smokers' hotline: Results of a 10-county self-help trial. Journal of consulting and clinical psychology. 1991;59(2):325.

174. Roski J, Jeddeloh R, An L, Lando H, Hannan P, Hall C, et al. The impact of financial incentives and a patient registry on preventive care quality: increasing provider adherence to evidence-based smoking cessation practice guidelines. Preventive medicine. 2003;36(3):291-9.

175. Bolman C, De Vries H, van Breukelen G. A minimal-contact intervention for cardiac inpatients: long-term effects on smoking cessation. Preventive medicine. 2002;35(2):181-92.

176. Borrelli B, Hayes RB, Dunsiger S, Fava JL. Risk perception and smoking behavior in medically ill smokers: a prospective study. Addiction. 2010;105(6):1100-8.

177. Hilberink SR, Jacobs JE, Bottema BJ, de Vries H, Grol RP. Smoking cessation in patients with COPD in daily general practice (SMOCC): six months' results. Preventive medicine. 2005;41(5):822-7.

178. Steptoe A, Day S, Doherty S, Rink E, Kerry S, Kendrick T, et al. Behavioural counselling in general practice for the promotion of healthy behaviour among adults at increased risk of coronary heart disease: randomised trialCommentary: Treatment allocation by the method of minimisation. Bmj. 1999;319(7215):943-8.

179. Wood D, Kotseva K, Connolly S, Jennings C, Mead A, Jones J. On behalf of EUROACTION Study Group Nurse-coordinated multidisciplinary, family-based cardiovascular disease prevention programme (EUROACTION) for patients with coronary heart disease and asymptomatic individuals at high risk of cardiovascular disease: a paired, cluster-randomised controlled trial. Lancet. 2008;371(9629):1999-2012.

180. Woodruff SI, Conway TL, Edwards CC, Elliott SP, Crittenden J. Evaluation of an Internet virtual world chat room for adolescent smoking cessation. Addictive behaviors. 2007;32(9):1769-86.

181. Baldwin N, Gilpin D, Tunney M, Kearney M, Crymble L, Cardwell C, et al. Cluster randomised controlled trial of an infection control education and training intervention programme focusing on meticillin-resistant Staphylococcus aureus in nursing homes for older people. Journal of Hospital Infection. 2010;76(1):36-41.

182. Houwing R, Van der Zwet W, van Asbeck S, Halfens R, Arends JW. An unexpected detrimental effect on the incidence of heel pressure ulcers after local 5% DMSO cream application: a randomized, double-blind study in patients at risk for pressure ulcers. WOUNDS-A COMPENDIUM OF CLINICAL RESEARCH AND PRACTICE. 2008;20(4):84-8.

183. Lui H, Hobbs L, Tope WD, Lee PK, Elmets C, Provost N, et al. Photodynamic Therapy of Multiple Nonmelanoma Skin Cancers With Verteporfinand Red Light–Emitting Diodes: Two-Year Results Evaluating Tumor Response and Cosmetic Outcomes. Archives of dermatology. 2004;140(1):26-32.

184. Morton C, Horn M, Leman J, Tack B, Bedane C, Tjioe M, et al. Comparison of topical methyl aminolevulinate photodynamic therapy with cryotherapy or fluorouracil for treatment of squamous cell carcinoma in situ: results of a multicenter randomized trial. Archives of dermatology. 2006;142(6):729-35.

185. Perrett C, McGregor J, Warwick J, Karran P, Leigh I, Proby C, et al. Treatment of post‐transplant premalignant skin disease: a randomized intrapatient comparative study of 5‐fluorouracil cream and topical photodynamic therapy. British Journal of Dermatology. 2007;156(2):320-8.

186. Salim A, Leman J, McColl J, Chapman R, Morton C. Randomized comparison of photodynamic therapy with topical 5‐fluorouracil in Bowen's disease. British Journal of Dermatology. 2003;148(3):539-43.

187. Aboulghar MM, Aboulghar MA, Amin YM, Al-Inany HG, Mansour RT, Serour GI. The use of vaginal natural progesterone for prevention of preterm birth in IVF/ICSI pregnancies. Reproductive biomedicine online. 2012;25(2):133-8.

188. Cetingoz E, Cam C, Sakallı M, Karateke A, Celik C, Sancak A. Progesterone effects on preterm birth in high-risk pregnancies: a randomized placebo-controlled trial. Archives of gynecology and obstetrics. 2011;283(3):423-9.

189. Combs CA, Garite T, Maurel K, Das A, Porto M. 17-hydroxyprogesterone caproate for twin pregnancy: a double-blind, randomized clinical trial. American Journal of Obstetrics and Gynecology. 2011;204(3):221.e1-.e8.

190. Elsheikhah AZ, Dahab S, Negm S, Ebrashy A, Momtaz M. OP19.08: Effect of prophylactic progesterone on incidence of preterm labour in spontaneous twin pregnancy, randomized controlled study. Ultrasound in Obstetrics & Gynecology. 2010;36(S1):108-.

191. Fonseca EB, Celik E, Parra M, Singh M, Nicolaides KH. Progesterone and the risk of preterm birth among women with a short cervix. New England Journal of Medicine. 2007;357(5):462-9.

192. Hartikainen-Sorri A-L, Kauppila A, Tuimala R. Inefficacy of 17 [alpha]-Hydroxyprogesterone Caproate in the Prevention of Prematurity in Twin Pregnancy. Obstetrics & Gynecology. 1980;56(6):692-5.

193. Norman JE, Mackenzie F, Owen P, Mactier H, Hanretty K, Cooper S, et al. Progesterone for the prevention of preterm birth in twin pregnancy (STOPPIT): a randomised, double-blind, placebo-controlled study and meta-analysis. The Lancet. 2009;373(9680):2034-40.

194. Rode L, Klein K, Nicolaides K, Krampl‐Bettelheim E, Tabor A. Prevention of preterm delivery in twin gestations (PREDICT): a multicenter, randomized, placebo‐controlled trial on the effect of vaginal micronized progesterone. Ultrasound in Obstetrics & Gynecology. 2011;38(3):272-80.

195. Rouse DJ, Caritis SN, Peaceman AM, Sciscione A, Thom EA, Spong CY, et al. A trial of 17 alpha-hydroxyprogesterone caproate to prevent prematurity in twins. New England Journal of Medicine. 2007;357(5):454-61.

196. Senat M-V, Porcher R, Winer N, Vayssière C, Deruelle P, Capelle M, et al. Prevention of preterm delivery by 17 alpha-hydroxyprogesterone caproate in asymptomatic twin pregnancies with a short cervix: a randomized controlled trial. American journal of obstetrics and gynecology. 2013;208(3):194. e1-. e8.

197. Serra V, Perales A, Meseguer J, Parrilla J, Lara C, Bellver J, et al. Increased doses of vaginal progesterone for the prevention of preterm birth in twin pregnancies: a randomised controlled double‐blind multicentre trial. BJOG: An International Journal of Obstetrics & Gynaecology. 2013;120(1):50-7.

198. Caritis SN, Rouse DJ, Peaceman AM, Sciscione A, Momirova V, Spong CY, et al. Prevention of Preterm Birth in Triplets: An Evaluation of 17 Alpha-Hydroxyprogesterone Caproate. Obstetrics and gynecology. 2009;113(2 Pt 1):285.

199. Combs CA, Garite T, Maurel K, Das A, Porto M, Network OCR. Failure of 17-hydroxyprogesterone to reduce neonatal morbidity or prolong triplet pregnancy: a double-blind, randomized clinical trial. American journal of obstetrics and gynecology. 2010;203(3):248. e1-. e9.

200. Lim AC, Schuit E, Bloemenkamp K, Bernardus RE, Duvekot JJ, Erwich JJH, et al. 17α-hydroxyprogesterone caproate for the prevention of adverse neonatal outcome in multiple pregnancies: a randomized controlled trial. Obstetrics & Gynecology. 2011;118(3):513-20.

201. Moore AA, Siu AL, Partridge JM, Hays RD, Adams J. A randomized trial of office-based screening for common problems in older persons. The American journal of medicine. 1997;102(4):371-8.
