# Supplementary material for: Cluster Randomised Trials in Cochrane Reviews: Evaluation of Methodological and Reporting Practice
Source: PLoS One. 2016 Mar 16;11(3):e0151818. doi: 10.1371/journal.pone.0151818 (PMC4794236; doi:10.1371/journal.pone.0151818)
Supplement: S1 Table — (DOCX) [file pone.0151818.s003.docx]

**Supplementary Table 1. Summary of included reviews**

| Review ID no. | Review | Cochrane Group | No. of trials | No. of C-RCTs | Types of studies | Types of patients | Types of interventions | Outcomes |
| --- | --- | --- | --- | --- | --- | --- | --- | --- |
| 1 | Antibiotics for preventing meningococcal infections | Cochrane Acute Respiratory Infections Group | 24 | 5 | Randomised controlled trials (RCTs) and quasi-RCTs | Healthy individuals:  exposed to someone with meningococcal disease, whether in the household or elsewhere  exposed to *N. meningitidis* carriers  belonging to a population with a high rate of *N. meningitidis* carriage, regardless of their carrier status | Intervention group: Drugs  *Interventions*  Antibiotic treatments  *Comparators*  Another antibiotic treatment  No intervention  Placebo | *Primary outcomes*   - Mortality - Occurrence of meningococcal infection   *Secondary outcomes*   - Occurrence of any clinical adverse effects - Proportion of meningococcal carriers and high-risk persons who were culture-negative at end of follow-up - Occurrence of relapse and re-colonisation - Occurrence of resistant strains subsequent to treatment |
| 2 | Influenza vaccination for healthcare workers who care for people aged 60 or older living in long-term care institutions | Cochrane Acute Respiratory Infections Group | 5 | 3 | RCTs and non-randomised controlled trials (N-RCTs) | Healthcare workers of all ages, caring for those aged 60 years or older in institutions such as nursing homes, LTCIs or hospital wards | Intervention group: Vaccines  *Interventions*  Vaccination of healthcare workers with any influenza vaccine given alone or with other vaccines, in any dose, preparation, or time schedule  *Comparators*  Placebo or no intervention | - Cases of influenza in those aged 60 years or older confirmed by viral isolation or serological supporting evidence (or both), plus a list of likely respiratory symptoms - Lower respiratory tract infection - Admission to hospital for respiratory illness - Deaths caused by respiratory illness |
| 3 | Integrated disease management interventions for patients with chronic obstructive pulmonary disease | Cochrane Airways Group | 26 | 2 | RCTs and cluster-RCTs (C-RCTs) | People with a clinical diagnosis of COPD according to the GOLD criteria | Intervention group: Non-pharmacological  *Interventions*  IDM programmes  *Comparators*  Usual care  No treatment  Single interventions  Mono-disciplinary interventions | *Primary outcomes*   - Health-related quality of life (HRQoL) - Maximal or functional exercise capacity - Exacerbation-related outcomes   *Secondary outcomes*  **Clinical outcomes**   - Dyspnea - Survival (mortality). - Lung function (FEV1, FVC). - Depression   **Process-related outcomes**   - Co-ordination of care, e.g. accessibility of care, participation rate in the disease management program, satisfaction of health care providers and participants with regard to the program, or the extent to which disease management was implemented, from the perspective of the patient and the caregiver |
| 4 | Physical conditioning as part of a return to work strategy to reduce sickness absence for workers with back pain | Cochrane Back Group | 25 | 2 | RCTs and C-RCTs | Male and female adults (> 16 years) with work disability related to back pain | Intervention group: Non-pharmacological  Physical conditioning programmes | - Time between intervention and return-to-work - Return-to-work status in terms of 'at work' or ‘off work’ - Time on light or modified duties. |
| 5 | Flexible sigmoidoscopy versus faecal occult blood testing for colorectal cancer screening in asymptomatic individuals | Cochrane Colorectal Cancer Group | 9 | 1 | RCTs | Adult (18 years and older) asymptomatic individuals | Intervention group: Screening  Flexible sigmoidoscopy versus CRC screening with FOBT | *Primary outcome*   - CRC mortality   *Secondary outcomes*   - CRC incidence - All-cause mortality - Attendance rates - Adverse effects - CRC staging - Use of endoscopy work-up |
| 6 | Mass media interventions for reducing mental health-related stigma | Cochrane Consumers and Communication Group | 22 | 3 | RCTs, C-RCTs, and interrupted time series (ITS) analyses | Members of the general public or any of its constituent groups (e.g. occupational or sociodemographic groups or any other target group), including children | Intervention group: Training/education based  *Interventions*  Mass media interventions on the subject of mental health  *Comparators*  Inactive control | *Primary outcomes*   - Discrimination towards people with mental ill health - Prejudice towards people with mental ill health   *Secondary outcomes*   - Knowledge (any type) - Cost of the mass media and comparator interventions - Reach, recall, and awareness of intervention(s) - Duration/sustainability of media effects - Audience reactions to media content - Unforeseen adverse effects |
| 7 | Interventions to promote informed consent for patients undergoing surgical and other invasive healthcare procedures | Cochrane Consumers and Communication Group | 65 | 2 | RCTs and C-RCTs | Patients aged 16 years and over being asked to give consent for a surgical or other invasive healthcare treatment or procedure, either for themselves, or on behalf of a minor or someone else for whom they have responsibility | Intervention group: Training/education based  Interventions with the intention of improving patients' understanding of their treatment options and the procedure under consideration, evaluating their options, or helping them retain and recall the information provided, and thus their ability to provide informed consent | *Primary outcome*   - Informed consent   *Secondary outcomes*   - Patient understanding - Knowledge/retention/recall - Deliberation (weighing up) - Communication of decision - Patient outcomes - Clinician outcomes - System outcomes |
| 8 | Enhanced care by generalists for functional somatic symptoms and disorders in primary care | Cochrane Depression, Anxiety and Neurosis Group | 7 | 7 | RCTs and C-RCTs | Studies will be limited to those involving adults (at least 18 years old) with FSS, identified either by case finding or by the primary care clinician’s assessment of FSS | Intervention group: Training/education based  *Intervention*  Specific training of participating clinicians; training focusing on the implementation of a model of enhanced care based on reattribution, reframing of symptoms, or characterised by psychosomatic explanations for physical symptoms and 'making the link' between symptoms and mental distress  *Comparators*  Usual care | *Primary outcome*   - Patient health status as measured by a validated quality of life tool   *Secondary outcomes*   - Measures of symptom load - The patients’ illness beliefs or illness worry (health anxiety) - Depression and anxiety - Functional status measured as sick leave - Patient satisfaction with care - Health care utilisation - Discontinuation of follow-up |
| 9 | Behavioural therapies versus other psychological therapies for depression | Cochrane Depression, Anxiety and Neurosis Group | 25 | 1 | RCTs, cross-over trials and C-RCTs | Men and women aged ≥ 18 years | Intervention group: Behavioural therapies  *Interventions*  Behavioural therapy  Behavioural activation  Social skills training/ assertiveness training  Relaxation therapy  Other behavioural therapies  Comparators  Cognitive-behavioural therapies (CBTs)  'Third wave' cognitive and behavioural therapies (third wave CBTs)  Psychodynamic therapies  Humanistic therapies  Interpersonal, cognitive analytic and other integrative therapies | *Primary outcomes*   - Treatment efficacy: the number of participants who responded to treatment, as determined by changes in validated depression scales - Treatment acceptability: the number of participants who dropped out of psychological therapy for any reason   *Secondary outcomes*   - The number of participants who remitted while receiving treatment - Improvement in depression symptoms - Improvement in overall symptoms - Improvement in anxiety symptoms - Adverse effects - Social adjustment and social functioning - Quality of life - Economic outcomes (e.g. days of work absence/ability to return to work, number of appointments with primary care physician, number of referrals to secondary services, use of additional treatments) |
| 10 | Ready-to-use therapeutic food for home-based treatment of severe acute malnutrition in children from six months to five years of age | Cochrane Developmental, Psychosocial and Learning Problems Group | 4 | 3 | RCTs, including quasi-randomised trials, and C-RCTs | Children between six months and five years of age with SAM, regardless of country, setting or disease status and irrespective of the method of diagnosis employed. | Intervention group: Non-pharmacological  *Interventions*  RUTF as defined by the study authors (either commercially or non-commercially produced).  *Comparators*  Alternative RUTF type (for example, corn/soy-based versus peanut-based, reduced milk powder content)  Treatment as usual (for example, standard diet) | *Primary outcomes*   - Recovery as defined by the study authors - Deterioration or relapse during and beyond the intervention period as defined by the study authors - Mortality   *Secondary outcomes*   - Mean weight gain per kilogram body weight per day during the intervention period - Time to recovery (duration to rehabilitation) - Anthropometrical status at all reported time points during and beyond the intervention period - Cognitive function and development during the intervention period - Adverse outcomes |
| 11 | Specially formulated foods for treating children with moderate acute malnutrition in low- and middle-income countries | Cochrane Developmental, Psychosocial and Learning Problems Group | 8 | 4 | RCTs, C-RCTs, quasi-randomised trials, N-RCTs, controlled before-and-after studies (CBAs), and ITS studies | Children in low- and middle-income countries aged 6 to 60 months with moderate acute malnutrition, treated either in hospital, a community clinic, or at home. | Intervention group: Non-pharmacological  *Interventions*  Improved adequacy of local diet  Lipid-based nutrient supplements (LNS)  Blended food supplements: corn-soy blended foods (CSB) or other blended foods such as as wheat-soy flour, sugar, oil, legumes, or others  Complementary food supplements  *Comparators*  Treatment as usual  Alternative food | *Primary outcomes*   - Recovered - Not recovered - Progression to severe acute malnutrition - Died - Defaulted (i.e. dropped out of the programme) - Weight gain - Weight-for-height - Mid-upper arm circumference - Adverse effects   *Secondary outcomes*   - Nutritional adequacy of the diet - Lean body mass increase - Height gain - Height-for-age - Coverage of the population |
| 12 | Educational and skills-based interventions for preventing relationship and dating violence in adolescents and young adults | Cochrane Developmental, Psychosocial and Learning Problems Group | 36 | 18 | RCTs, C-RCTs and quasi-RCTs | Adolescents aged 12 to 18 years and young adults aged 19 to 25 years in any setting | Intervention group: Training/education-based  Any programme that was applied universally or to specifically targeted high-risk groups and actively provided adolescents or young adults with educational or skills-based interventions, or both, aimed at the prevention of dating or relationship violence | *Primary outcomes*   - Reduction in the number of episodes of relationship and dating violence experienced - Reduction in injuries resulting from relationship and dating violence experienced - Self-reported subjective improvement in mental well-being - Adverse events (i.e. an increase in the number of episodes of relationship or dating violence, or both)   *Secondary outcomes*   - Improvements in behaviour or knowledge about relationship and dating violence (participant-reported) - Improvements in access to (or knowledge of) help or support services (participant-reported) - Attainment of protective skills (participant-reported) - Intervention-related factors: cost of the programme, time commitment required and acceptability of the programme (as measured by dropout rate) |
| 13 | Non-specialist health worker interventions for the care of mental, neurological and substance-abuse disorders in low- and middle-income countries | Cochrane Effective Practice and Organisation of Care Group | 38 | 10 | RCTs, N-RCTs, CBA studies and ITS studies | Children (aged below 18 years) or adults with any MNS seeking first-level care/primary care or who were detected in the community in LMICs, and carers of people with MNS disorders | Intervention group: Drugs/Psychosocial/Non-Pharmacological  Clinical (medical and psychological) and service interventions delivered in primary care or the community by NSHWs or OPHRs, and intended to improve MNS disorders | *Primary outcomes*   - Improvement of symptoms (e.g. level of anxiety, depression, psychosis) - Psychosocial functioning and impairment (e.g. levels of self-esteem, perception of coping, level of dependency, self-care ability) - Quality of life outcomes (including disability)   *Secondary outcomes*  For studies evaluating the detection of mental disorders and the delivery of acute and chronic mental health interventions:   **Patient/carer-oriented outcomes and societal outcomes**   - Patient or carer satisfaction and involvement in decision-making processes - Patient health behaviour outcomes: such as rates of patient adherence or treatment/follow-up compliance, utilisation of primary level services - Adverse clinical outcomes: such as adverse effects rates, suicide/deliberate self-harm rates, relapse or recurrence, hospital admission/readmission rates - Patient social outcomes: return to work, offending rates, perception of social inclusion - Carer outcomes: such as mental health outcomes, quality of life and functioning   **Health provider and service delivery related outcomes**   - Measures of changes in management (such as referral rates, prescribing patterns and appropriateness) - Measures of health worker behaviour (such as improvement in knowledge/skills, attitude/acceptability, retention rates, absenteeism) - Measures of service delivery change (such as number of supervision sessions, effect on other health services provided)     For studies of costs and resource use:   - Direct and indirect costs to the patient and health services (including opportunity costs) - Resource use (such as the patient's lost productivity, and health service personnel's time allocated/number of consultations) |
| 14 | Computerized advice on drug dosage to improve prescribing practice | Cochrane Effective Practice and Organisation of Care Group | 42 | 5 | RCTs, C-RCTs, N-RCTs, CBA studies and ITS studies | Healthcare professionals with responsibility for patient care | Intervention group: Counselling/advice  *Intervention*  Computerized advice on drug dosage given  *Comparators*  Routine care (empiric dosing without computer assistance) | - Proportion of participants or time for which the plasma drug concentrations was within the therapeutic range - Proportion of participants or time for which the studied physiological parameter was maintained within the target range - Time to achieve therapeutic control - Proportion of participants with toxic drug levels - Proportion of participants with clinical improvement - Proportion of participants with adverse effects of drug therapy - Proportion of deaths - Length of hospital stay - Total cost per participant |
| 15 | The effect of different methods of remuneration on the behaviour of primary care dentists | Cochrane Effective Practice and Organisation of Care Group | 2 | 2 | RCTs, N-RCTs, CBA studies (at least two sites in each group) and ITS studies | Primary care dentists providing routine dental care in primary care environments | Intervention group: Non-pharmacological  Fee-for-service payments  Fixed salary payments  Capitation payments  Blended payments (combinations of above) | *Primary outcomes*   - Measures of clinical activity - Measures of health service utilisation - Healthcare costs - Patient outcomes   *Secondary outcomes*   - Measures of non-clinical behaviour of primary care dentists including the rates of performing specified non-clinical behaviours (e.g. education and training), when specified as a secondary outcome. - Measures of dental practice profitability/income. - Any unintended effects of the remuneration systems, including supplier-induced demand when the service provided is not based on need, changes to the types of treatment offered, and limitations to access. |
| 16 | Behavioral interventions for improving condom use for dual protection | Cochrane Fertility Regulation Group | 7 | 6 | Randomized or non-randomized studies | Heterosexual women or heterosexual men. Participants may have been at risk for pregnancy or HIV/STI and could be HIV-positive or HIV-negative. | Intervention group: Behavioural therapies  Behavioral interventions addressing the use of condoms specifically, that is, with an educational or counselling component to encourage or improve condom use | - Pregnancy (test result or birth record) - HIV (test result) - Sexually transmitted infection (test result) - Presence of semen as assessed with a biological marker |
| 17 | Theory-based interventions for contraception | Cochrane Fertility Regulation Group | 17 | 7 | RCTs and C-RCTs | Women who were users or potential users of the contraceptive methods | Intervention group: Non-pharmacological  Interventions addressing the use of one or more contraceptive methods for contraception. Any hormonal or non-hormonal contraceptive could have been studied, such as oral contraceptives or intrauterine devices. Theory-based interventions, based on for e.g., theories or models of education, communication, or behavior change. | *Primary outcomes*   - Pregnancy (test or self-report) - Contraceptive choice - Initiation of, or change in, contraceptive use - Adherence to contraceptive regimen - Contraceptive continuation   *Secondary outcomes*   - Knowledge of contraceptive effectiveness - Attitude about contraception in general or about a specific contraceptive method |
| 18 | Remote and web 2.0 interventions for promoting physical activity | Cochrane Heart Group | 11 | 1 | RCTs | Community dwelling adults, aged from 16 years to any age, who were free from pre-existing medical conditions or with no more than 10% of participants with pre-existing medical conditions that may have limited participation in PA. | Intervention group: Counselling/advice  Remote and web 2.0 PA interventions that included:  counselling or advice, or both;  self-directed or prescribed exercise, or both;  home based or facility based exercise, or both;  written education or motivational support material, or both.  The comparison was with a control group exposed to placebo or no or minimal intervention. | *Primary outcomes*   - Cardio-respiratory fitness - PA levels   *Secondary outcomes*   - Quality of life (for example quality-adjusted life years (QALYs)) - Cost (for example cost-benefit, cost-utility) - Adverse events (for example musculoskeletal injury, cardiovascular event) |
| 19 | Decentralising HIV treatment in lower- and middle-income countries | Cochrane HIV/AIDS Group | 16 | 2 | RCTs, N-RCTs, and CBA studies.  Prospective and retrospective cohort studies with a comparison between standard and decentralised delivery | HIV-infected patients at the point of initiating treatment, and patients already on treatment requiring maintenance and follow-up | Intervention group: Non-pharmacological  *Interventions*  Any form of decentralised care delivery model for the initiation of treatment, continuation of treatment, or both.  *Comparator*s  Care delivered at the centralised site (usually a hospital, or in the case of community interventions, any facility) | *Primary outcomes*   - Attrition, defined as a composite of loss to follow-up or death - Loss to follow-up at set time points after the intervention has been introduced, as defined by the study authors - Death, after being considered eligible for treatment, or during treatment   *Secondary outcomes*   - Time to starting antiretroviral treatment - Patients diagnosed with tuberculosis after entry into HIV care - Virologic response to ART - Immunologic response to ART - Occurrence of a new AIDS-defining illness - Patient satisfaction with care - Cost to the provider - Cost to the patient and family - Any negative impact on other programme and health care delivery reported by the authors |
| 20 | Primaquine for preventing relapse in people with *Plasmodium vivax*malaria treated with chloroquine | Cochrane Infectious Diseases Group | 15 | 2 | RCTs and quasi-RCTs. | Adults and children with microscopically confirmed asexual *P. vivax* malaria | Intervention group: Drugs  Interventions  Primaquine (any dose or duration other than used in control group) plus chloroquine  *Comparators*  Primaquine (15 mg/day for 14 days) plus chloroquine  Placebo or no intervention plus chloroquine | *Primary outcomes*   - *P. vivax* parasitaemia detected more than 30 days after starting primaquine. - Serious adverse events (fatal, life threatening, or requiring hospitalization)   *Secondary outcomes*   - Adverse events that result in the discontinuation of treatment - Events known to occur with primaquine (cyanosis, leucopenia, methaemoglobinaemia, hypertension, cardiac arrhythmia, abdominal pain, nausea, vomiting, and haemolysis) or those due to a comparator drug used along with primaquine - Other adverse events |
| 21 | Mosquito larval source management for controlling malaria | Cochrane Infectious Diseases Group | 13 | 4 | C-RCTs, CBA studies for which the unit of allocation was the cluster, cross-over trials for which the unit of randomization was the cluster | Children and adults living in rural and urban malaria-endemic areas | Intervention group: Non-pharmacological  Interventions aimed to reduce the emergence of adult vectors from aquatic habitats | *Primary outcomes*   - Incidence of malaria: diagnostically confirmed by rapid diagnostic test or microscopy - Parasite prevalence: diagnostically confirmed by rapid diagnostic test or microscopy     *Secondary outcomes*   - Splenomegaly prevalence in children - Anaemia prevalence in children - Time to infection - Total mortality of children aged under five years - EIR: the estimated number of bites by infectious mosquitoes per person per unit time - Adult mosquito density |
| 22 | Rifamycins (rifampicin, rifabutin and rifapentine) compared to isoniazid for preventing tuberculosis in HIV-negative people at risk of active TB | Cochrane Infectious Diseases Group | 10 | 2 | RCTs and C-RCTs | HIV-negative people at risk of developing active TB and without active TB at the time of enrolmen | Intervention group: Drugs  *Intervention*  Treatment with rifampicin or rifamycin-containing drug combinations (any dose or duration)    *Comparator*  INH monotherapy for six to 12 months. | *Primary outcome*   - Rates of active TB   *Secondary outcomes*   - TB-related deaths - All-cause death - Incidence of drug-resistant TB including MDR-TB and XDR-TB - Adherence to treatment - Adverse events |
| 23 | Screening for lung cancer | Cochrane Lung Cancer Group | 9 | 1 | RCTs, C-RCTs and controlled clinical trials (CCTs) | Adults from all backgrounds including men and women, smokers, non-smokers and ex-smokers | Intervention groups: Screening  Chest x-ray, computed tomography (CT), sputum cytology or other sputum examinations, alone or in any possible combination or frequency | *Primary outcome*  Disease-specific mortality  *Other outcomes*   - Compliance with screening and follow up - Incidence of lung cancer - Five-year survival - Stage at diagnosis - Resection rate - Postoperative deaths - Harms of screening including adverse outcomes from further diagnostic testing in those who have a positive result on initial screening - Costs - All-cause mortality - Quality of life |
| 24 | Targeting intensive glycaemic control versus targeting conventional glycaemic control for type 2 diabetes mellitus | Cochrane Metabolic and Endocrine Disorders Group | 28 | 1 | All randomised clinical trials of any design | Adults aged 18 years and above with T2D were included | Intervention group: Drugs  *Intervention*  Intensive glycaemic control    *Comparator*  Conventional glycaemic control | *Primary outcomes*   - All-cause mortality - Cardiovascular mortality (death from myocardial infarction, stroke, and peripheral vascular disease)   *Secondary outcomes*   - Macrovascular complications (non-fatal myocardial infarction, non-fatal ischaemic stroke, non-fatal haemorrhagic stroke, amputation of lower extremity, and cardiac or peripheral revascularization) - Microvascular complications (manifestation and progression of nephropathy, end-stage renal disease, manifestation and progression of retinopathy, and retinal photocoagulation) - Adverse events (number of patients with any untoward medical occurrence not necessarily having a causal relationship with the treatment) - Congestive heart failure - Hypoglycaemia - Health-related quality of life measured with validated instruments - Cost(s) of intervention |
| 25 | Non-pharmacological interventions for fatigue in rheumatoid arthritis | Cochrane Musculoskeletal Group | 24 | 1 | RCTs | Adults (usually over 18 years of age) with a diagnosis of RA either confirmed by a rheumatologist or using American College of Rheumatology (ACR) criteria | Intervention group: Non-pharmacological  All non-pharmacological interventions, which encompassed all interventions other than those that were classified as pharmacological | *Primary outcome*   - Self-reported fatigue scores using validated measures, and adverse events   *Secondary outcomes*   - Pain, anxiety, depression, disability and tender or swollen joints |
| 26 | Cycled light in the intensive care unit for preterm and low birth weight infants | Cochrane Neonatal Group | 8 | 1 | RCTs, C-RCTs and quasi-RCTs | Preterm infants (< 37 weeks' PMA or low birth weight < 2500 g) admitted and cared for in a NICU or a stepdown unit | Intervention group: Non-pharmacological  CL versus irregularly DL or ND or CBL initiated during hospitalisation in the NICU | *Primary outcome*   - Growth at three and six months' CA (g/day, or actual weight)   *Secondary outcomes*   - Time to full oral or nasogastric feeds (days) - Chronic lung disease (CLD) or bronchopulmonary dysplasia (BPD) (oxygen requirement above 0.21 at 28 days' and 36 weeks' PMA) - Days on assisted ventilation - Days in oxygen above 0.21 - ROP; any stage and stages ≥3 - Days of initial hospitalisation - Long-term outcomes: growth and neurodevelopmental including visual and auditory outcome at any age - Any clinically important outcome not listed above but reported by the authors - Carers' satisfaction or dissatisfaction with the intervention - Parents' satisfaction or dissatisfaction with the intervention - Adverse effects |
| 27 | Enamel etching for bonding fixed orthodontic braces | Cochrane Oral Health Group | 13 | 13 | RCTs | Children and adults, with fixed orthodontic brackets attached to their incisors, canines and premolars | Intervention group: Techniques  Any kind of etching materials and techniques used in the intervention group compared with different etching materials, times and acid concentrations, or an alternative etching technique in the control group | *Primary outcome*   - Bond failure rate of braces (the rate at which braces fall off the teeth during treatment)   *Secondary outcomes*   - The presence or absence of decay (decalcification) associated with or around the etching field - Participant satisfaction - Cost of treatment |
| 28 | Screening programmes for the early detection and prevention of oral cancer | Cochrane Oral Health Group | 1 | 1 | RCTs | Participants involved in population, selective (high-risk) or opportunistic screening programmes | Intervention group: Screening  Visual screening;  Visual staining using toluidine blue;  Oral cytology using brush biopsies;  Fluorescence imaging and light-based techniques. | *Primary outcome*   - Oral cancer mortality   Other outcomes   - Incidence of oral cancer or PMD - Stage at diagnosis - Adverse effects - Cost data |
| 29 | Fluoride varnishes for preventing dental caries in children and adolescents | Cochrane Oral Health Group | 22 | 5 | RCTs and quasi-RCTs | Children or adolescents aged 16 or less at the start of the study (irrespective of initial level of dental caries, background exposure to fluorides, dental treatment level, nationality, setting where intervention is received or time when it started) | Intervention group: Non-pharmacological  *Intervention*  Fluoride varnish  *Comparator*  Placebo or no treatment | *Primary outcome*   - Caries increment, as measured by change from baseline in the number of decayed, (missing) and filled permanent surfaces / number of decayed, (extracted/missing) and filled primary surfaces   *Other outcomes*   - Coronal dental caries and dental fillings, in both the permanent and the primary dentitions, tooth loss, dental pain, specific adverse effects, use of health service resources (such as visits to dental care units, length of dental treatment time) |
| 30 | Effectiveness and cost-effectiveness of home palliative care services for adults with advanced illness and their caregivers | Cochrane Pain, Palliative and Supportive Care Group | 23 | 3 | RCTs, C-RCTs, patient or cluster CCTs  CBA studies and ITS analyses | Participants aged 18 years or older in receipt of a home palliative care service (as described below), their family caregivers, or both | Intervention group: Non-pharmacological  A team delivering home palliative care with the presence of the following four elements:  1. Primarily for patients with a severe or advanced disease (malignant or non-malignant), no longer responding to curative/maintenance treatment or symptomatic (or both), or their family caregivers, or both  2. Aiming to support patients or family caregivers, or both, outside hospital and other institutional settings as far as possible and to enable patients to stay at home  3. Providing either specialist or intermediate palliative/hospice care  4. Providing comprehensive care and aiming at different physical and psychosocial components of palliative care | *Primary outcome*  Death at home  *Secondary outcomes*   - Time the patient spent at home - Satisfaction with care - Pain - Other symptoms - Physical function - Quality of life - Caregiver pre- and post-bereavement outcomes |
| 31 | Interventions for implementation of thromboprophylaxis in hospitalized medical and surgical patients at risk for venous thromboembolism | Cochrane Peripheral Vascular Diseases Group | 55 | 7 | RCTs, C-RCTs, quasi-RCTs, and non-randomized studies (NRS) with or without concurrent controls | Hospitalized adult medical or surgical inpatients | Intervention group: Non-pharmacological  Any strategies targeted to individuals or to clusters aimed to increase the use of thromboprophylaxis in hospitalized patients at risk for VTE and/or decrease the rate of symptomatic or asymptomatic VTE | *Primary outcome*   - Proportion of patients who received prophylaxis (RP) or received appropriate prophylaxis (RAP)   *Secondary outcomes*   - Reduction in the proportion of symptomatic VTE - Reduction in the proportion of asymptomatic VTE - Safety of the intervention, e.g. frequency of clinically relevant bleeding (major hemorrhage; minor hemorrhage) or other complications |
| 32 | Fetal and umbilical Doppler ultrasound in high-risk pregnancies | Cochrane Pregnancy and Childbirth Group | 18 | 3 | All randomised trials and quasi-randomised studies | Women with pregnancies considered to be at 'high risk' for fetal compromise, e.g. intrauterine growth restriction, post-term pregnancies, previous pregnancy loss, women with hypertension, women with diabetes or other maternal pathology (e.g. thrombophilia) | Intervention group: Screening  Doppler ultrasound of the fetal and umbilical vessels for fetal assessment in pregnancies in high-risk populations | *Primary outcomes*   - Any perinatal death after randomisation - Serious neonatal morbidity - composite outcome including hypoxic ischaemic encephalopathy, intraventricular haemorrhage (IVH), bronchopulmonary dysplasia (BPD), necrotising enterocolitis (NEC)   *Secondary outcomes*   - Stillbirth - Neonatal death - Any potentially preventable perinatal death - Fetal acidosis - Apgar score less than seven at five minutes - Caesarean section (both elective and emergency) - Spontaneous vaginal birth - Operative vaginal birth - Induction of labour - Oxytocin augmentation - Neonatal resuscitation required - Infant requiring intubation/ventilation - Neonatal fitting/seizures - Preterm labour - Gestational age at birth - Infant respiratory distress syndrome - Meconium aspiration - Neonatal admission to special care or intensive care unit, or both - Hypoxic ischaemic encephalopathy (a condition of injury to the brain) - IVH - BPD - NEC - Infant birthweight - Length of infant hospital stay - Long-term infant/child neurodevelopmental outcome - Women's views of their care |
| 33 | Psychosocial interventions for supporting women to stop smoking in pregnancy | Cochrane Pregnancy and Childbirth Group | 44 | 16 | RCTs, C-RCTs, cross-over randomised trials, and quasi-randomised studies (only if there was a very low risk of interference with the sequence generation) | Women who are currently smoking or have recently quit smoking and are pregnant, in any care setting.  Women who are currently smoking or have recently quit smoking and are seeking a pre-pregnancy consultation.  Health professionals in trials of implementation strategies of psychosocial interventions to support pregnant women to stop smoking. | Intervention groups: Psychosocial  *Interventions*  Counselling interventions  Health education interventions  Feedback interventions  Incentive-based interventions  Social support (peer and/or partner)  Other strategies  *Comparators*  'Usual care'  Less intensive interventions  Alternative interventions | *Primary outcomes*   - Smoking abstinence in late pregnancy (point prevalence abstinence)   *Secondary outcomes*   - Continued abstinence in late pregnancy after spontaneous quitting (relapse prevention) in early pregnancy (self-reported or biochemically validated) - Smoking abstinence in the postpartum period (self-reported or biochemically validated)   1. zero to five months;   2. six to 11 months;   3. 12 to 17 months;   4. 18 months or longer. - Smoking reduction from the first antenatal visit to late pregnancy:   1. numbers of women reducing smoking (any definition, > 50% self-reported, or biochemically validated);   2. biochemical measures (mean cotinine and thiocynate);   3. mean cigarettes per day (self-reported). - Perinatal outcomes:   1. mean birthweight;   2. low birthweight (proportion less than 2500 g);   3. very low birthweight (less than 1500 g);   4. preterm births (proportion less than 37 weeks);   5. stillbirths;   6. neonatal deaths;   7. all perinatal deaths. - Mode of birth (caesarean section) - Breastfeeding initiation and breastfeeding at three and six months after birth - Psychological effects: measures of anxiety, depression and maternal health status in late pregnancy and after birth - Impact on family functioning and other relationships in late pregnancy and postpartum - Participants' views of the interventions, both women’s and pregnancy care providers’ views - Measures of knowledge, attitudes and behaviour of health professionals (obstetricians, midwives and family physicians) with respect to facilitating smoking cessation in pregnancy - Cost-effectiveness - Adverse effects of smoking cessation programmes |
| 34 | Midwife-led continuity models versus other models of care for childbearing women | Cochrane Pregnancy and Childbirth Group | 13 | 1 | RCTs, C-RCTs and trials where allocation was alternate or not clear | Pregnant women | Intervention group: Non-pharmacological  *Interventions*  Midwife-led continuity models of care, i.e. the midwife is the woman's lead professional, but one or more consultations with medical staff are often part of routine practice  *Comparators*  Other models of care including:  a) where the physician/obstetrician is the lead professional, and midwives and/or nurses provide intrapartum care and in-hospital postpartum care under medical supervision  b) shared care, where the lead professional changes depending on whether the woman is pregnant, in labour or has given birth, and on whether the care is given in the hospital, birth centre (free standing or integrated) or in community setting(s)  c) where the majority of care is provided by physicians or obstetricians. | *Primary outcomes*  **Birth and immediate postpartum**  Regional analgesia (epidural/spinal)  Caesarean birth  Instrumental vaginal birth (forceps/vacuum)  Spontaneous vaginal birth (as defined by trial authors)  Intact perineum    **Neonatal**  Preterm birth (less than 37 weeks)  Overall fetal loss and neonatal death (fetal loss was assessed by gestation using 24 weeks as the cut-off for viability in many countries)  *Secondary outcomes*   - Antenatal hospitalisation - Antepartum haemorrhage - Induction of labour - Amniotomy - Augmentation/artificial oxytocin during labour - No intrapartum analgesia/anaesthesia - Opiate analgesia - Attendance at birth by known midwife - Episiotomy - Perineal laceration requiring suturing - Mean labour length (hours) - Postpartum haemorrhage - Breastfeeding initiation - Duration of postnatal hospital stay (days) - Low birthweight (less than 2500 g) - Five-minute Apgar score less than or equal to seven - Neonatal convulsions - Admission to special care nursery/neonatal intensive care unit - Mean length of neonatal hospital stay (days) - Fetal loss and neonatal death less than 24 weeks - Fetal loss and neonatal death equal to/after 24 weeks - Perceptions of control during labour and childbirth - Mean number of antenatal visits - Maternal death - Cord blood acidosis - Postpartum depression - Any breastfeeding at three months - Prolonged perineal pain - Pain during sexual intercourse - Urinary incontinence - Faecal incontinence - Prolonged backache |
| 35 | Schedules for home visits in the early postpartum period | Cochrane Pregnancy and Childbirth Group | 12 | 3 | RCTs, C-RCTs, and quasi-RCTs | Participants were in the early postpartum period (up to 42 days after birth) | Intervention group: Non-pharmacological  Scheduled home visiting in the postpartum period (excluding studies with antenatal home visiting in which the visits continued over many months). Interventions were home visits with various frequency, timings, duration and intensity. | *Primary outcomes*   - Maternal mortality at 42 days post birth - Neonatal mortality   *Secondary outcomes*  **Maternal outcomes**   - Maternal morbidities (postpartum haemorrhage, puerperal fever, abdominal and back pain, abnormal discharge, puerperal genital infection, thromboembolic disease, and urinary tract complications) within 42 days after birth - Maternal mental health (depression, anxiety) and related problems (intimate partner violence, drug use) at 42 days after birth - Satisfaction with overall care and service at 42 days after birth     **Neonatal outcomes**   - Neonatal morbidities (pneumonia, upper respiratory tract infection, diarrhoea, septic meningitis, encephalopathy or cerebral injury, and jaundice) within 28 days after birth - Established feeding regimen (e.g. exclusive breastfeeding) at 28 days after birth. - Incomplete immunisation - Failure to thrive, abuse, neglect, domestic violence from parents for any reason within 28 days after birth |
| 36 | Telephone support for women during pregnancy and the first six weeks postpartum | Cochrane Pregnancy and Childbirth Group | 29 | 1 | RCTs and C-RCTs | Pregnant women and postnatal women in the first six weeks post birth | Intervention group: Non-pharmacological  *Interventions*  All interventions aimed at supporting women by using telephones, whether for general support/information or for a specific medical/social reason (e.g. diabetes, smoking)  *Comparators*  Any other supportive intervention  No telephone support | *Primary outcomes*   - Maternal satisfaction with support during pregnancy and the first six months postpartum (as defined by trial authors) - Maternal anxiety (measures as defined by trial authors, e.g. Hospital Anxiety and Depression Scale).   *Secondary outcomes*  **Maternal outcomes**   - Mother-infant attachment. - General health (e.g. as defined by standardised measures such as general health questionnaires) - Mortality and serious morbidity (e.g. perineal haematoma or deep surgical infection) - Health service utilisation (presentation/attendance at clinics, accident and emergency departments or general practices) - Postpartum depression (measures as defined by author, e.g. the Edinburgh Postnatal Depression Scale (EPDS)) - Positive behaviour change (as defined by trial authors, e.g. smoking reduction)     **Infant outcomes**   - Preterm birth/low birthweight - Breastfeeding duration (exclusive or combined feeding) - Infant developmental measures (physical and cognitive as defined by trial authors) - Neonatal/infant mortality - Major neonatal/infant morbidity (as defined by trial authors, e.g. prolonged admission to special care baby unit)     **Service**   - Intervention cost |
| 37 | Interventions to improve water quality and supply, sanitation and hygiene practices, and their effects on the nutritional status of children | Cochrane Public Health Group | 17 | 5 | RCTs, C-RCTs, quasi-RCTs, N-RCTs, CBA studies (cohort or cross-sectional), ITS and historically controlled studies. | Children aged under 18 years | Intervention group: Non-pharmacological  Interventions  Any intervention aimed at improving the microbiological quality of drinking water  Any intervention aimed at introducing a new or improved water supply or improved distribution, or both.  Interventions aimed at introducing or expanding the coverage and use of facilities designed to improve sanitation.  Interventions aimed at the promotion of handwashing with soap after defecation, disposal of child faeces and prior to preparing and handling food.  Any combination of the WASH interventions listed above.    Comparators  Water quality: study participants who have continued with usual practice, or a less stringent version of the intervention (i.e. new protected well but no household disinfection on top of this).  Water supply: study participants who have continued with usual practice.  Sanitation: study participants who have continued to practice open defecation or who continue with usual practice regarding excreta disposal rather than following the prescribed intervention.  Hygiene: no handwashing promotion; study participants who continued with usual practice. | *Primary outcome*   - Child nutritional status as measured by anthropometry: weight-for-age (underweight), weight-for-height (wasting), height-for-age (stunting)   *Secondary outcomes*   - Child nutritional status as measured by anthropometry: weight, height, mid-upper arm circumference, skinfold thickness, percent body fat, birthweight, body mass index (BMI) - Child nutritional status as measured by nutrient status: haemoglobin, serum ferritin, soluble transferrin receptor, serum retinol, serum zinc, urinary iodine, clinical signs of nutrient deficiency |
| 38 | User-held personalised information for routine care of people with severe mental illness | Cochrane Schizophrenia Group | 4 | 2 | RCTs | Adults with severe mental illness (SMI) defined as diagnosis of a psychotic illness, including other psychoses such as bipolar disorder and depression with psychotic features | Intervention group: Non-pharmacological  User-held information: any personalised and accessible clinical information held by the patient beyond standard care.  Standard information: any information routinely held such as appointment cards and generic information on diagnosis, treatment or services available. | *Primary outcomes*   - Psychiatric hospital admission - Death from causes other than suicide - Violence   *Secondary outcomes*   - Rates of criminal charges - Mental state  1. Relapse of psychotic illness 2. Mental state score  - Satisfaction with health care  1. Patient satisfaction 2. Carer satisfaction  - Perceived coercion on hospital admission - Acceptability of management (as measured by loss to follow-up within the study) - Compliance with treatment other than the intervention - Social functioning  1. Homelessness 2. Employment 3. Average change in social functioning  - Economic costs of all care and health care |
| 39 | Tobacco cessation interventions for young people | Cochrane Tobacco Addiction Group | 28 | 7 | RCTs, C-RCTs, N-RCTs | Participants aged less than 20, who are regular tobacco smokers (smokes an average of at least one cigarette a week, and has done so for at least six months) | Intervention group: Drugs/non-pharmacological  Interventions were aimed at helping young people to stop smoking tobacco, including pharmacotherapy, targeting individual young people, through strategic programmes targeting people or organizations associated with young people (for example, their families or schools), and complex programmes targeting the community in which young people study or live | *Primary outcome*   - Smoking status at six months follow-up or longer   *Secondary outcome*   - Adverse events |
| 40 | Smoking cessation interventions for smokers with current or past depression | Cochrane Tobacco Addiction Group | 49 | 1 | RCTs | Adult smokers with current or past depression | Intervention group: Drugs/psychosocial  Any pharmacological or psychosocial intervention, or a combination of both, to aid smoking cessation in smokers with current or past depression | *Primary outcome*   - Smoking status at a minimum of six months from the quit day |
| 41 | Relapse prevention interventions for smoking cessation | Cochrane Tobacco Addiction Group | 63 | 9 | RCTs and quasi-RCTs | People who had quit smoking on their own; people who were undergoing enforced abstinence, whether or not they intended to quit permanently; and smokers participating in treatment programmes to assist initial cessation | Intervention group: Drugs/psychosocial/behavioural  *Interventions*  Interventions were intended to prevent relapse:  Behavioural interventions delivered in any format, including group meetings, face-to-face sessions, written or other materials, proactive or reactive telephone support  Pharmacological interventions  *Comparators*  No intervention or a shorter intervention or an intervention not oriented towards relapse prevention | *Primary outcome*   - Prolonged or multiple point prevalence abstinence at follow-up of at least six months since randomization   *Other outcomes*   - Point prevalence abstinence (number of participants not smoking at the point when assessment is made-abstinent at that time but not necessarily continuously since treatment) at six months or longer |
| 42 | Telephone counselling for smoking cessation | Cochrane Tobacco Addiction Group | 77 | 11 | RCTs, C-RCTs and quasi-RCTs | Smokers or recent quitters | Intervention group: Counselling/advice  Provision of proactive or reactive telephone counselling to assist smoking cessation, to any population | *Primary outcome*   - Smoking cessation at least six months after the start of intervention |
| 43 | Nursing interventions for smoking cessation | Cochrane Tobacco Addiction Group | 49 | 6 | RCTs | Participants were adult smokers, 18 years and older, of either gender and recruited in any type of healthcare or other setting | Intervention group: Counselling/advice  Nursing intervention was defined as the provision of advice, counselling, and/or strategies to help people quit smoking. | *Primary outcome*   - Smoking cessation with a follow-up period of at least 6 months |
| 44 | Internet-based interventions for smoking cessation | Cochrane Tobacco Addiction Group | 28 | 1 | RCTs or quasi-RCTs | Smokers who participated in Internet interventions for smoking cessation, with no exclusions on the basis of age, gender, ethnicity, language spoken or health status | Intervention group: Non-pharmacological  Internet interventions in all settings and from all types of providers | *Primary outcome*   - Smoking cessation at least six months after the start of the intervention   *Other outcomes*   - User satisfaction rates |
| 45 | Infection control strategies for preventing the transmission of meticillin-resistant *Staphylococcus aureus* (MRSA) in nursing homes for older people | Cochrane Wounds Group | 1 | 1 | RCTs, C-RCTs, N-RCTs, CBA studies and ITS analyses (with at least three points before and after the intervention) | Residents over the age of 65 years, living in nursing homes | Intervention group: Non-pharmacological  *Interventions*  Barrier precautions including screening, individual isolation, patient cohort isolation, nurse cohorting, use of gloves, aprons and face masks.  Hand washing including the use of antiseptics with water or hand washing with alcohol gel in the absence of water.  Environmental hygiene including cleaning, disinfection and sterilisation procedures  *Comparators*  Usual care | *Primary outcome*   - MRSA incidence   *Secondary outcomes*   - MRSA prevalence - All-cause mortality - Length of hospital stay - Rates of antibiotic therapy - Quality of life (e.g. effect of isolating residents for prolonged periods of time) |
| 46 | Dressings and topical agents for preventing pressure ulcers | Cochrane Wounds Group | 9 | 1 | RCTs and C-RCTs | People of any age, both adults and children, without a pressure ulcer, but considered to be at risk of developing a pressure ulcer, in any care setting | Intervention group: Non-pharmacological  The primary intervention was any wound dressing or topical agent applied to the skin at any frequency with the aim of preventing the development of a pressure ulcer | *Primary outcome*   - Pressure ulcer incidence (the proportion of people developing any new pressure ulcer(s) of any grade)   *Secondary outcomes*   - Stage of any new pressure ulcer(s) - Time to ulcer development - Costs of interventions - Quality of life as measured by a validated scale - Pain at dressing change, measured using a validated scale - Acceptability of the intervention (or satisfaction) with respect to patient comfort - Adverse events - Length of hospital stay |
| 47 | Interventions for cutaneous Bowen's disease | Cochrane Skin Group | 9 | 4 | RCTs | All adults with histologically proven cutaneous Bowen's disease | Intervention group: Techniques  Any interventions for the treatment of cutaneous Bowen's disease, including:  Surgical  Destructive  Other techniques | *Primary outcome*   - Complete clearance of the lesion:  1. Number of lesions or participants cleared after first treatment cycle 2. Recurrence at 12 months   *Secondary outcomes*   - Number of lesions that cleared after each treatment cycle - Number of treatment cycles needed to achieve clearance - Recurrence at > 12 months - Cosmetic outcome using a recognised and validated instrument to measure cosmesis - Consumer satisfaction with treatment modality, cosmesis, or pain at site, recorded on a Likert or Likert-like scale - Time to complete healing of lesion following treatment - Quality of life - Adverse outcomes - Recurrence of Bowen's disease in same site determined by clinical examination |
| 48 | Beta-lactam versus beta-lactam-aminoglycoside combination therapy in cancer patients with neutropenia | Cochrane Gynaecological Cancer Group | 71 | 23 | RCTs and quasi-RCTs | Febrile cancer patients with neutropenia, as defined in the study, induced by chemotherapy or bone marrow transplantation | Intervention group: Drugs  *Interventions*  Intravenous beta-lactam antibiotic given as monotherapy  Combination duotherapy of an intravenous beta-lactam antibiotic (as specified) with one of the following aminoglycosides given intravenously: gentamicin, tobramycin, amikacin, netilmicin, kanamycin | *Primary outcome*   - Death at end of follow-up for the infectious episode, up to 30 days (all-cause mortality)   *Secondary outcomes*   - Treatment failure: a composite end point comprising one or more of the following: death; persistence, recurrence or worsening of clinical signs or symptoms of presenting infection; any modification of the assigned empirical antibiotic treatment - Infection related mortality, as reported in the study - Duration of hospital stay - Dropouts before end of study - Super-infection: new, persistent or worsening symptoms and/or signs of infection associated with the isolation of a new pathogen (different, or different susceptibilities) or the development of a new site of infection - Colonisation: isolation during or after therapy of Gram-negative bacteria resistant to the beta-lactam included in the empirical regimen, without symptoms or signs of infection - Adverse effects |
| 49 | Prenatal administration of progesterone for preventing preterm birth in women considered to be at risk of preterm birth | Cochrane Pregnancy and Childbirth Group | 36 | 14 | RCTs | Pregnant women considered to be at increased risk of preterm birth | Intervention group: Drugs  Administration of progesterone by any route for the prevention of preterm birth | *Primary outcomes*   - Perinatal mortality - Preterm birth (less than 34 weeks' gestation) - Major neurodevelopmental handicap at childhood follow-up   *Secondary outcomes*  **Maternal**   - Threatened preterm labour (as defined by trial authors) - Prelabour spontaneous rupture of membranes - Adverse drug reaction - Pregnancy prolongation (interval between randomisation and birth) - Mode of birth - Number of antenatal hospital admissions - Satisfaction with the therapy - Use of tocolysis - Antenatal corticosteroids (not a prespecified outcome) - Maternal quality of life (not a prespecified outcome)     **Infant**   - Birth before 37 completed weeks - Birth before 28 completed weeks - Birthweight less than the third centile for gestational age - Birthweight less than 2500 g - Apgar score of less than seven at five minutes - Respiratory distress syndrome - Use of mechanical ventilation - Duration of mechanical ventilation - Intraventricular haemorrhage - grades III or IV - Periventricular leucomalacia - Retinopathy of prematurity - Retinopathy of prematurity - grades III or IV - Chronic lung disease - Necrotising enterocolitis - Neonatal sepsis - Fetal death - Neonatal death - Admission to neonatal intensive care unit - Neonatal length of hospital stay - Teratogenic effects (including virilisation in female infants) - Patent ductus arteriosis (not a prespecified outcome)     **Child**   - Major sensorineural disability (defined as any of legal blindness, sensorineural deafness requiring hearing aids, moderate or severe cerebral palsy, or developmental delay or intellectual impairment (defined as developmental quotient or intelligence quotient less than -2 standard deviations below mean)) - Developmental delay (however defined by the authors) - Intellectual impairment - Motor impairment - Visual impairment - Blindness - Deafness - Hearing impairment - Cerebral palsy - Child behaviour - Child temperament - Learning difficulties - Growth assessments at childhood follow-up (weight, head circumference, length, skin fold thickness) |
| 50 | Nutritional screening for improving professional practice for patient outcomes in hospital and primary care settings | Cochrane Pain, Palliative and Supportive Care Group | 3 | 1 | RCTs, C-RCTs, cross-over designs, block randomised designs, N-RCTs, CBA studies and ITS analyses | Participants were adult patients aged 16 years or over, in hospital, an out-patient clinic, primary care or long term care, and also individuals that were otherwise considered healthy but might be at risk of under-nutrition | Intervention group: Screening  Interventions are screening tools for a group of patients, or otherwise healthy people, for whom the level of under-nutrition risk is unknown to establish whether they are at under-nutrition risk | *Primary outcomes*   - Patient outcomes: mortality, morbidity (e.g. wound infection), health related quality of life (measured by validated generic or condition specific instruments)   *Secondary outcomes*   - Patient outcomes e.g. change in body mass index (BMI) or weight - Process outcomes: identification of patients requiring nutritional care, data recording (e.g. weight and BMI), referral of patients to dietitians or similar, nutritional interventions, dietary advice to patients, direct medical costs, hospitalisation, hospital length of stay - Adverse outcomes |

CBA=controlled before-after; CCT=controlled clinical trial; C-RCT=cluster-randomised controlled trial; ITS=interrupted time series; N-RCT=non-randomised controlled trial; NRS=non-randomised study; RCT=randomised controlled trial
