# Supplementary material for: Cluster Randomised Trials in Cochrane Reviews: Evaluation of Methodological and Reporting Practice
Source: PLoS One. 2016 Mar 16;11(3):e0151818. doi: 10.1371/journal.pone.0151818 (PMC4794236; doi:10.1371/journal.pone.0151818)
Supplement: S3 Table — (DOCX) [file pone.0151818.s005.docx]

Supplementary Table 3. Assessment of “Reporting C-RCTs”

| Review | Cochrane Group | Trial (reference corresponding to citations in S7 File) | Trial report obtained? | Is unit of randomisation reported for each C-RCT? | Is study design i.e. matched pairs, stratified, reported for each C-RCT? | Is it reported whether the trial is adjusted or unadjusted for clustering for each C-RCT? | Is method of cluster adjustment reported for each C-RCT? | Is intra-cluster correlation coefficient for each C-RCT for each outcome? | Is average cluster size reported for each C-RCT? |
| --- | --- | --- | --- | --- | --- | --- | --- | --- | --- |
| Antibiotics for preventing meningococcal infections | Cochrane Acute Respiratory Infections Group | Blakebrough and Gilles (1) | Y | Y | Y | N | NR (in trial report) | NR (in trial report) | NR (in trial report) |
|  |  | Cuevas, Kazembe (2) | Y | Y | Y | N | NR (in trial report) | NR (in trial report) | NR (in trial report) |
|  |  | Guttler, Counts (3) | Y | Y | Y | N | NR (in trial report) | NR (in trial report) | N |
|  |  | Munford, Sussuarana de Vasconcelos (4) | Y | Y | Y | N | NR (in trial report) | NR (in trial report) | N |
|  |  | Schwartz (5) | Y | Y | Y | N | NR (in trial report) | NR (in trial report) | NR (in trial report) |
| Influenza vaccination for healthcare workers who care for people aged 60 or older living in long-term care institutions | Cochrane Acute Respiratory Infections Group | Carman, Elder (6) | Y | Y | Y | Y | N/A | NR (in trial report) | Y |
|  |  | Potter, Stott (7) | Y | Y | Y | Y | Y | NR (in trial report) | Y |
|  |  | Lemaitre, Meret (8) | Y | Y | Y | Y | Y | NR (in trial report) | Y |
| Integrated disease management interventions for patients with chronic obstructive pulmonary disease | Cochrane Airways Group | Rea, McAuley (9) | Y | Y | Y | Y | N/A | NR (in trial report) | N |
|  |  | Wood-Baker, McGlone (10) | Y | Y | Y | Y | N/A | NR (in trial report) | N |
| Physical conditioning as part of a return to work strategy to reduce sickness absence for workers with back pain | Cochrane Back Group | Bethge, Herbold (11) | Y | N | Y | N | N | N | N |
|  |  | Loisel, Abenhaim (12) | Y | Y | N | N | NR (in trial report) | NR (in trial report) | N |
| Flexible sigmoidoscopy versus faecal occult blood testing for colorectal cancer screening in asymptomatic individuals | Cochrane Colorectal Cancer Group | Segnan, Armaroli (13) | Y | Y | N | Y | N/A | NR (in trial report) | N |
| Mass media interventions for reducing mental health-related stigma | Cochrane Consumers and Communication Group | Coleman (14) | Y | Y | Y | Y | N/A | NR (in trial report) | NR (in trial report) |
|  |  | Penn, Chamberlin (15) | Y | Y | Y | Y | N/A | NR (in trial report) | Y |
|  |  | Yoshida (NTR) | N | NTR | NTR | NTR | NTR | NTR | NTR |
| Interventions to promote informed consent for patients undergoing surgical and other invasive healthcare procedures | Cochrane Consumers and Communication Group | Solberg, Asche (16) | Y | Y | Y | N | N | NR (in trial report) | Y |
|  |  | Paci, Barneschi (17) | Y | Y | Y | N | NR (in trial report) | NR (in trial report) | NR (in trial report) |
| Enhanced care by generalists for functional somatic symptoms and disorders in primary care | Cochrane Depression, Anxiety and Neurosis Group | Blankenstein (18) | Y | Y | N | Y | N/A | NR (in trial report) | Y |
|  |  | Larisch, Schweickhardt (19) | Y | Y | Y | Y | N | NR (in trial report) | Y |
|  |  | Morriss, Dowrick (20) | Y | Y | Y | Y | N | NR (in trial report) | Y |
|  |  | Rief, Martin (21) | Y | Y | Y | Y | N/A | NR (in trial report) | Y |
|  |  | Rosendal, Olesen (22) | Y | Y | N | Y | N | N | Y |
|  |  | Toft, Rosendal (23) | Y | Y | Y | Y | N | NR (in trial report) | Y |
|  |  | Whitehead and Campion (24) | Y | Y | Y | Y | N/A | NR (in trial report) | NR (in trial report) |
| Behavioural therapies versus other psychological therapies for depression | Cochrane Depression, Anxiety and Neurosis Group | Rude (25) | Y | Y | Y | N | NR (in trial report) | NR (in trial report) | Y |
| Ready-to-use therapeutic food for home-based treatment of severe acute malnutrition in children from six months to five years of age | Cochrane Developmental, Psychosocial and Learning Problems Group | Ciliberto, Sandige (26) | Y | N | Y | Y | N/A | NR (in trial report) | N |
|  |  | Manary, Ndkeha (27) | Y | Y | Y | Y | N/A | NR (in trial report) | N |
|  |  | Ndekha, Manary (28) | Y | Y | Y | Y | N/A | NR (in trial report) | NR (in trial report) |
| Specially formulated foods for treating children with moderate acute malnutrition in low- and middle-income countries | Cochrane Developmental, Psychosocial and Learning Problems Group | Ackatia-Armah, McDonald (29) | Y | Y | Y | Y | Y | NR (in trial report) | Y |
|  |  | Delchevalerie (NTR) | N | NTR | NTR | NTR | NTR | NTR | NTR |
|  |  | Karakochuk, van den Briel (30) | Y | Y | Y | Y | Y | NR (in trial report) | Y |
|  |  | Nikiema (NTR) | N | NTR | NTR | NTR | NTR | NTR | NTR |
| Educational and skills-based interventions for preventing relationship and dating violence in adolescents and young adults | Cochrane Developmental, Psychosocial and Learning Problems Group | Andersen (31) | Y | Y | Y | Y | N/A | NR (in trial report) | Y |
|  |  | Anderson, Stoelb (32) | Y | Y | Y | Y | N/A | NR (in trial report) | N |
|  |  | Avery-Leaf, Cascardi (33) | Y | Y | Y | Y | N/A | NR (in trial report) | NR (in trial report) |
|  |  | Boulter (34) | Y | Y | Y | Y | N/A | NR (in trial report) | Y |
|  |  | Breitenbecher and Gidycz (35) | Y | Y | Y | Y | N/A | NR (in trial report) | Y |
|  |  | Fay and Medway (36) | Y | Y | Y | Y | N/A | NR (in trial report) | Y |
|  |  | Foshee, Bauman (37) | Y | Y | Y | Y | N/A | NR (in trial report) | Y |
|  |  | Foubert and Marriott (38) | Y | Y | Y | Y | N/A | NR (in trial report) | N |
|  |  | Foubert and McEwen (39) | Y | Y | Y | Y | N/A | NR (in trial report) | Y |
|  |  | Foubert (40) | Y | Y | Y | Y | N/A | NR (in trial report) | Y |
|  |  | Gidycz, Orchowski (41) | Y | Y | Y | Y | N/A | NR (in trial report) | Y |
|  |  | Holcomb, Savage (42) | Y | Y | Y | Y | N/A | NR (in trial report) | N |
|  |  | Jaycox, McCaffrey (43) | Y | Y | Y | N | N | NR (in trial report) | N |
|  |  | Macgowan (44) | Y | Y | Y | Y | N/A | NR (in trial report) | N |
|  |  | Miller, Tancredi (45) | Y | Y | Y | N | N | NR (in trial report) | Y |
|  |  | Pacifici, Stoolmiller (46) | Y | Y | Y | Y | N/A | NR (in trial report) | Y |
|  |  | Saberi (47) | Y | Y | Y | Y | N/A | NR (in trial report) | Y |
|  |  | Wolfe, Crooks (48) | Y | Y | Y | Y | Y | Y | Y |
| Non-specialist health worker interventions for the care of mental, neurological and substance-abuse disorders in low- and middle-income countries | Cochrane Effective Practice and Organisation of Care Group | Baker-Henningham, Powell (49) | Y | Y | Y | N | N | NR (in trial report) | N |
|  |  | Berger and Gelkopf (50) | Y | Y | Y | Y | N/A | NR (in trial report) | N |
|  |  | Bolton, Bass (51) | Y | Y | Y | Y | N | NR (in trial report) | N |
|  |  | Hirani, Karmaliani (52) | Y | Y | Y | N | NR (in trial report) | NR (in trial report) | Y |
|  |  | Jenkins, Othieno (53) | Y | Y | Y | Y | N | N | N |
|  |  | Jordans, Komproe (54) | Y | Y | Y | Y | N | NR (in trial report) | Y |
|  |  | Patel, Weiss (55) | Y | Y | Y | N | N | Y | N |
|  |  | Rahman, Malik (56) | Y | Y | Y | N | NR (in trial report) | N | N |
|  |  | Tol, Komproe (57) | Y | Y | Y | Y | N | N | N |
|  |  | Tol, Komproe (58) | Y | Y | Y | N | N | N | N |
| Computerized advice on drug dosage to improve prescribing practice | Cochrane Effective Practice and Organisation of Care Group | Burton, Ash (59) | Y | Y | Y | Y | N/A | NR (in trial report) | Y |
|  |  | Fitzmaurice, Hobbs (60) | Y | Y | N | Y | NR (in trial report) | NR (in trial report) | Y |
|  |  | Claes, Buntinx (61) | Y | Y | Y | Y | N | NR (in trial report) | Y |
|  |  | Terrell, Perkins (62) | Y | Y | Y | Y | N | NR (in trial report) | Y |
|  |  | Wexler, Shrader (63) | Y | Y | Y | Y | N/A | NR (in trial report) | Y |
| The effect of different methods of remuneration on the behaviour of primary care dentists | Cochrane Effective Practice and Organisation of Care Group | Clarkson, Turner (64) | Y | Y | Y | Y | Y | Y | Y |
|  |  | Coventry, Holloway (65) | Y | Y | Y | Y | N/A | NR (in trial report) | Y |
| Behavioral interventions for improving condom use for dual protection | Cochrane Fertility Regulation Group | Kamali, Kinsman (66) | Y | Y | Y | N | NR (in trial report) | N | Y |
|  |  | Ross, Changalucha (67) | Y | Y | Y | Y | Y | N | Y |
|  |  | Jewkes, Nduna (68) | Y | Y | Y | Y | Y | N | Y |
|  |  | Cowan, Pascoe (69) | Y | Y | Y | Y | Y | N | Y |
|  |  | Boyer, Shafer (70) | Y | Y | Y | Y | Y | N | N |
|  |  | Stephenson, Strange (71) | Y | Y | Y | Y | Y | N | Y |
| Theory-based interventions for contraception | Cochrane Fertility Regulation Group | Coyle, Basen-Engquist (72) | Y | Y | Y | Y | Y | NR (in trial report) | Y |
|  |  | Wight, Raab (73) | Y | Y | Y | Y | Y | N | Y |
|  |  | Stanton, Cole (74) | Y | Y | Y | Y | Y | N | Y |
|  |  | Boyer, Shafer (70) | Y | Y | Y | Y | Y | N | NR (in trial report) |
|  |  | Coyle, Kirby (75) | Y | Y | Y | Y | Y | NR (in trial report) | Y |
|  |  | Ross, Changalucha (67) | Y | Y | Y | Y | Y | NR (in trial report) | Y |
|  |  | Cowan, Pascoe (69) | Y | Y | Y | Y | Y | NR (in trial report) | Y |
| Remote and web 2.0 interventions for promoting physical activity | Cochrane Heart Group | Elley, Kerse (76) | Y | Y | N | N | N | N | N |
| Decentralising HIV treatment in lower- and middle-income countries | Cochrane HIV/AIDS Group | Jaffar, Amuron (77) | Y | Y | N | Y | N/A | Y | Y |
|  |  | Selke, Kimaiyo (78) | Y | Y | Y | Y | N/A | NR (in trial report) | N |
| Primaquine for preventing relapse in people with *Plasmodium vivax*malaria treated with chloroquine | Cochrane Infectious Diseases Group | Leslie, Rab (79) | Y | Y | Y | Y | N | NR (in trial report) | N |
|  |  | Leslie, Mayan (80) | Y | Y | Y | Y | N | NR (in trial report) | NR (in trial report) |
| Mosquito larval source management for controlling malaria | Cochrane Infectious Diseases Group | Yapabandara, Curtis (81) | Y | Y | N | Y | N/A | NR (in trial report) | Y |
|  |  | Yapabandara and Curtis (82) | Y | Y | N | Y | N/A | NR (in trial report) | Y |
|  |  | Shililu, Mbogo (83) | Y | Y | Y | Y | N/A | NR (in trial report) | NR (in trial report) |
|  |  | Coulibaly (NTR) | N | NTR | NTR | NTR | NTR | NTR | NTR |
| Rifamycins (rifampicin, rifabutin and rifapentine) compared to isoniazid for preventing tuberculosis in HIV-negative people at risk of active TB | Cochrane Infectious Diseases Group | Tortajada, Martinez-Lacasa (84) | Y | Y | Y | Y | N | NR (in trial report) | NR (in trial report) |
|  |  | Sterling, Villarino (85) | Y | Y | Y | Y | N/A | NR (in trial report) | NR (in trial report) |
| Screening for lung cancer | Cochrane Lung Cancer Group | Brett (86) | Y | Y | Y | Y | N/A | NR (in trial report) | N |
| Targeting intensive glycaemic control versus targeting conventional glycaemic control for type 2 diabetes mellitus | Cochrane Metabolic and Endocrine Disorders Group | Griffin, Borch-Johnsen (87) | Y | Y | N | Y | N | Y | N |
| Non-pharmacological interventions for fatigue in rheumatoid arthritis | Cochrane Musculoskeletal Group | Häkkinen, Sokka (88) | Y | Y | Y | N | NR (in trial report) | NR (in trial report) | Y |
| Cycled light in the intensive care unit for preterm and low birth weight infants | Cochrane Neonatal Group | Brandon, Holditch-Davis (89) | Y | N | Y | N | NR (in trial report) | NR (in trial report) | NR (in trial report) |
| Enamel etching for bonding fixed orthodontic braces | Cochrane Oral Health Group | Aljubouri, Millett (90) | Y | Y | Y | Y | Y | NR (in trial report) | Y |
|  |  | Asgari, Salas (91) | Y | Y | Y | Y | N/A | NR (in trial report) | Y |
|  |  | Banks and Thiruvenkatachari (92) | Y | Y | Y | Y | Y | NR (in trial report) | Y |
|  |  | e Cal-Neto, Quintão (93) | Y | Y | Y | Y | N/A | NR (in trial report) | Y |
|  |  | Elekdag-Turk, Isci (94) | Y | Y | Y | Y | Y | NR (in trial report) | Y |
|  |  | Elekdag-Turk, Cakmak (95) | Y | Y | Y | Y | Y | NR (in trial report) | Y |
|  |  | Ghiz, Ngan (96) | Y | Y | Y | Y | N/A | NR (in trial report) | Y |
|  |  | House, Ireland (97) | Y | Y | Y | Y | N/A | NR (in trial report) | Y |
|  |  | Ireland, Knight (98) | Y | Y | Y | Y | N/A | NR (in trial report) | Y |
|  |  | Manning, Chadwick (99) | Y | Y | Y | Y | Y | NR (in trial report) | Y |
|  |  | Murfitt, Quick (100) | Y | Y | Y | Y | N/A | NR (in trial report) | Y |
|  |  | Noble, Salas-Lopez (101) | Y | Y | Y | Y | N/A | NR (in trial report) | Y |
|  |  | Paschos, Kurochkina (102) | Y | Y | Y | Y | N/A | NR (in trial report) | Y |
| Screening programmes for the early detection and prevention of oral cancer | Cochrane Oral Health Group | Sankaranarayanan, Ramadas (103) | Y | Y | Y | Y | N/A | NR (in trial report) | Y |
| Fluoride varnishes for preventing dental caries in children and adolescents | Cochrane Oral Health Group | Borutta, Reuscher (104) | Y | Y | Y | N | N/A | NR (in trial report) | Y |
|  |  | Bravo, Baca (105) | Y | Y | Y | Y | N/A | NR (in trial report) | Y |
|  |  | Hardman, Davies (106) | Y | Y | Y | Y | N/A | N | Y |
|  |  | Lawrence, Binguis (107) | Y | Y | Y | Y | N | Y | Y |
|  |  | Milsom, Blinkhorn (108) | Y | Y | Y | Y | NR (in trial report) | N | N |
| Effectiveness and cost-effectiveness of home palliative care services for adults with advanced illness and their caregivers | Cochrane Pain, Palliative and Supportive Care Group | Jordhøy, Fayers (109) | Y | Y | Y | N | N | NR (in trial report) | Y |
|  |  | McKegney, Bailey (110) | Y | Y | Y | N | NR (in trial report) | NR (in trial report) | NR (in trial report) |
|  |  | Rabow, Dibble (111) | Y | Y | Y | N | NR (in trial report) | NR (in trial report) | Y |
| Interventions for implementation of thromboprophylaxis in hospitalized medical and surgical patients at risk for venous thromboembolism | Cochrane Peripheral Vascular Diseases Group | Anderson, Wheeler (112) | Y | Y | N | Y | N/A | NR (in trial report) | N |
|  |  | Dexter, Perkins (113) | Y | Y | Y | Y | N/A | NR (in trial report) | N |
|  |  | Fontaine, Mahe (114) | Y | Y | Y | Y | N/A | NR (in trial report) | N |
|  |  | Garcia, Highfill (115) | Y | Y | Y | Y | N/A | NR (in trial report) | N |
|  |  | Labarere, Bosson (116) | Y | Y | Y | Y | N | N | N |
|  |  | Overhage, Tierney (117) | Y | Y | Y | Y | N | NR (in trial report) | N |
| Fetal and umbilical Doppler ultrasound in high-risk pregnancies | Cochrane Pregnancy and Childbirth Group | Giles, Bisits (118) | Y | Y | Y | Y | N/A | NR (in trial report) | Y |
|  |  | Johnstone, Prescott (119) | Y | Y | Y | Y | N/A | NR (in trial report) | Y |
|  |  | Newnham, O'Dea (120) | Y | Y | Y | Y | N/A | NR (in trial report) | Y |
| Psychosocial interventions for supporting women to stop smoking in pregnancy | Cochrane Pregnancy and Childbirth Group | Bauman, Bryan (121) | Y | NR (in trial report) | NR (in trial report) | Y | N/A | NR (in trial report) | NR (in trial report) |
|  |  | Campbell, Walsh (122) | Y | Y | Y | N | N/A | NR (in trial report) | Y |
|  |  | Eades, Sanson-Fisher (123) | Y | Y | Y | Y | n/a | NR (in trial report) | NR (in trial report) |
|  |  | Hajek, West (124) | Y | Y | Y | Y | N/A | NR (in trial report) | Y |
|  |  | Haug, Fugelli (125) | Y | Y | Y | N | N/A | NR (in trial report) | Y |
|  |  | Kendrick, Zahniser (126) | Y | Y | Y | Y | N | Y | Y |
|  |  | Lawrence, Aveyard (127) | Y | Y | Y | Y | N | Y | Y |
|  |  | Lillington, Royce (128) | Y | Y | Y | Y | N/A | NR (in trial report) | Y |
|  |  | Lowe, Balanda (129) | Y | Y | Y | N | NR (in trial report) | NR (in trial report) | NR (in trial report) |
|  |  | Manfredi, Crittenden (130) | Y | Y | Y | N | N | NR (in trial report) | Y |
|  |  | McLeod, Pullon (131) | Y | Y | Y | Y | N | NR (in trial report) | Y |
|  |  | Messimer, Hickner (132) | Y | Y | N | N | N/A | NR (in trial report) | Y |
|  |  | Moore, Campbell (133) | Y | Y | Y | Y | N | Y | Y |
|  |  | Pbert, Ockene (134) | Y | Y | Y | Y | N | NR (in trial report) | Y |
|  |  | Polańska, Hanke (135) | Y | Y | Y | Y | N | NR (in trial report) | Y |
|  |  | Vilches (NTR) | NTR | NTR | NTR | NTR | NTR | NTR | NTR |
| Midwife-led continuity models versus other models of care for childbearing women | Cochrane Pregnancy and Childbirth Group | North Staffordshire Changing Childbirth Research Team (136) | Y | N | Y | Y | Y | Y | N |
| Schedules for home visits in the early postpartum period | Cochrane Pregnancy and Childbirth Group | Christie and Bunting (137) | Y | Y | Y | Y | N | NR (in trial report) | N |
|  |  | Kronborg, Væth (138) | Y | Y | N | Y | N | NR (in trial report) | Y |
|  |  | MacArthur, Winter (139) | Y | Y | Y | Y | N | NR (in trial report) | Y |
| Telephone support for women during pregnancy and the first six weeks postpartum | Cochrane Pregnancy and Childbirth Group | Lund, Hemed (140) | Y | Y | Y | Y | N | NR (in trial report) | Y |
| Interventions to improve water quality and supply, sanitation and hygiene practices, and their effects on the nutritional status of children | Cochrane Public Health Group | Du Preez, McGuigan (141) | Y | Y | Y | N | N | N | Y |
|  |  | Du Preez, Conroy (142) | Y | Y | Y | N | N | N | Y |
|  |  | Luby, Agboatwalla (143) | Y | Y | Y | N | N | NR (in trial report) | N |
|  |  | Luby, Agboatwalla (144) | Y | Y | Y | N | N | N | N |
|  |  | McGuigan, Samaiyar (145) | Y | Y | Y | N | N | N | Y |
| User-held personalised information for routine care of people with severe mental illness | Cochrane Schizophrenia Group | Warner, King (146) | Y | Y | Y | Y | N/A | Y | N |
|  |  | Lester, Allan (147) | Y | Y | Y | Y | N | N | Y |
| Tobacco cessation interventions for young people | Cochrane Tobacco Addiction Group | Aveyard, Cheng (148) | Y | Y | Y | N | N | N | Y |
|  |  | Chan and Witherspoon (149) | Y | Y | Y | N | N/A | NR (in trial report) | Y |
|  |  | Hoffman, Nemes (150) | Y | Y | Y | N | N | NR (in trial report) | Y |
|  |  | Horn, Dino (151) | Y | Y | Y | N | N/A | NR (in trial report) | Y |
|  |  | Idrisov, Sun (152) | Y | Y | Y | N | N | NR (in trial report) | N |
|  |  | McCuller, Sussman (153) | Y | Y | Y | N | N/A | NR (in trial report) | Y |
|  |  | Woodruff, Conway (154) | Y | Y | Y | Y | N/A | NR (in trial report) | Y |
| Smoking cessation interventions for smokers with current or past depression | Cochrane Tobacco Addiction Group | Batra, Collins (155) | Y | N | N | N | N | NR (in trial report) | N |
| Relapse prevention interventions for smoking cessation | Cochrane Tobacco Addiction Group | Conway, Woodruff (156) | Y | Y | Y | N | N/A | NR (in trial report) | NR (in trial report) |
|  |  | Hajek, West (124) | Y | Y | Y | N | N/A | NR (in trial report) | N |
|  |  | Klesges, Haddock (157) | Y | Y | Y | N | N/A | Y | N |
|  |  | Klesges, DeBon (158) | Y | Y | Y | N | N | N | N |
|  |  | Mayer, Vandecasteele (159) | Y | Y | Y | N | N/A | NR (in trial report) | Y |
|  |  | Mermelstein, Hedeker (160) | Y | Y | Y | N | N/A | NR (in trial report) | N |
|  |  | Pbert, Ockene (134) | Y | Y | Y | N | N | NR (in trial report) | Y |
|  |  | Razavi, Vandecasteele (161) | Y | Y | Y | N | N/A | NR (in trial report) | N |
|  |  | Schröter, Collins (162) | Y | Y | Y | N | N/A | NR (in trial report) | Y |
|  |  | Severson, Andrews (163) | Y | Y | Y | Y | N | N | Y |
| Telephone counselling for smoking cessation | Cochrane Tobacco Addiction Group | Borland, Balmford (164) | Y | Y | Y | Y | N | N | Y |
|  |  | Chouinard and Robichaud-Ekstrand (165) | Y | Y | Y | N | N/A | NR (in trial report) | Y |
|  |  | Ebbert, Carr (166) | Y | Y | Y | N | N/A | NR (in trial report) | Y |
|  |  | Hennrikus, Jeffery (167) | Y | Y | Y | N | N | NR (in trial report) | Y |
|  |  | Joyce, Niaura (168) | Y | Y | Y | N | N | NR (in trial report) | Y |
|  |  | Katz, Muehlenbruch (169) | Y | Y | N | N | N | N | Y |
|  |  | Lando, Rolnick (170) | Y | Y | Y | N | N | NR (in trial report) | N |
|  |  | Lichtenstein, Andrews (171) | Y | Y | Y | Y | N/A | Y | Y |
|  |  | Lichtenstein, Boles (172) | Y | Y | Y | N | N/A | NR (in trial report) | Y |
|  |  | Ossip-Klein, Giovino (173) | Y | Y | Y | Y | Y | NR (in trial report) | Y |
|  |  | Roski, Jeddeloh (174) | Y | Y | N | N | N | NR (in trial report) | Y |
| Nursing interventions for smoking cessation | Cochrane Tobacco Addiction Group | Bolman, De Vries (175) | Y | Y | Y | N | N | N | Y |
|  |  | Borrelli, Hayes (176) | Y | Y | Y | Y | N/A | NR (in trial report) | Y |
|  |  | Chouinard and Robichaud-Ekstrand (165) | Y | Y | Y | N | N/A | NR (in trial report) | Y |
|  |  | Hilberink, Jacobs (177) | Y | Y | N | Y | Y | N | Y |
|  |  | Steptoe, Day (178) | Y | Y | Y | N | N | N | Y |
|  |  | Wood, Kotseva (179) | Y | Y | N | N | N | N | N |
| Internet-based interventions for smoking cessation | Cochrane Tobacco Addiction Group | Woodruff, Conway (180) | Y | Y | Y | Y | N/A | Y | Y |
| Infection control strategies for preventing the transmission of meticillin-resistant *Staphylococcus aureus* (MRSA) in nursing homes for older people | Cochrane Wounds Group | Baldwin, Gilpin (181) | Y | Y | Y | Y | N | Y | Y |
| Dressings and topical agents for preventing pressure ulcers | Cochrane Wounds Group | Houwing, Van der Zwet (182) | Y | Y | Y | Y | N/A | N | NR (in trial report) |
| Interventions for cutaneous Bowen's disease | Cochrane Skin Group | Lui, Hobbs (183) | Y | Y | Y | Y | N/A | NR (in trial report) | Y |
|  |  | Morton, Horn (184) | Y | Y | Y | Y | N/A | NR (in trial report) | Y |
|  |  | Perrett, McGregor (185) | Y | Y | Y | Y | N/A | NR (in trial report) | Y |
|  |  | Salim, Leman (186) | Y | Y | Y | Y | N/A | NR (in trial report) | Y |
| Beta-lactam versus beta-lactam-aminoglycoside combination therapy in cancer patients with neutropenia | Cochrane Gynaecological Cancer Group | Review stated there were 23 included C-RCTs, it was unclear which of the 71 included trials were classified as C-RCTs. Contacted author for more information but classification of C-RCTs was still unclear | N | NTR | NTR | NTR | NTR | NTR | NTR |
|  |  |  | N | NTR | NTR | NTR | NTR | NTR | NTR |
|  |  |  | N | NTR | NTR | NTR | NTR | NTR | NTR |
|  |  |  | N | NTR | NTR | NTR | NTR | NTR | NTR |
|  |  |  | N | NTR | NTR | NTR | NTR | NTR | NTR |
|  |  |  | N | NTR | NTR | NTR | NTR | NTR | NTR |
|  |  |  | N | NTR | NTR | NTR | NTR | NTR | NTR |
|  |  |  | N | NTR | NTR | NTR | NTR | NTR | NTR |
|  |  |  | N | NTR | NTR | NTR | NTR | NTR | NTR |
|  |  |  | N | NTR | NTR | NTR | NTR | NTR | NTR |
|  |  |  | N | NTR | NTR | NTR | NTR | NTR | NTR |
|  |  |  | N | NTR | NTR | NTR | NTR | NTR | NTR |
|  |  |  | N | NTR | NTR | NTR | NTR | NTR | NTR |
|  |  |  | N | NTR | NTR | NTR | NTR | NTR | NTR |
|  |  |  | N | NTR | NTR | NTR | NTR | NTR | NTR |
|  |  |  | N | NTR | NTR | NTR | NTR | NTR | NTR |
|  |  |  | N | NTR | NTR | NTR | NTR | NTR | NTR |
|  |  |  | N | NTR | NTR | NTR | NTR | NTR | NTR |
|  |  |  | N | NTR | NTR | NTR | NTR | NTR | NTR |
|  |  |  | N | NTR | NTR | NTR | NTR | NTR | NTR |
|  |  |  | N | NTR | NTR | NTR | NTR | NTR | NTR |
|  |  |  | N | NTR | NTR | NTR | NTR | NTR | NTR |
|  |  |  | N | NTR | NTR | NTR | NTR | NTR | NTR |
| Prenatal administration of progesterone for preventing preterm birth in women considered to be at risk of preterm birth | Cochrane Pregnancy and Childbirth Group | Aboulghar, Aboulghar (187) | Y | Y | Y | Y | N/A | NR (in trial report) | Y |
|  |  | Cetingoz, Cam (188) | Y | Y | Y | Y | N/A | NR (in trial report) | Y |
|  |  | Combs, Garite (189) | Y | Y | Y | Y | Y | NR (in trial report) | Y |
|  |  | Elsheikhah, Dahab (190) | Y | Y | Y | Y | N/A | NR (in trial report) | Y |
|  |  | Fonseca, Celik (191) | Y | Y | Y | Y | Y | NR (in trial report) | Y |
|  |  | Hartikainen-Sorri, Kauppila (192) | Y | Y | Y | Y | N/A | NR (in trial report) | Y |
|  |  | Norman, Mackenzie (193) | Y | Y | Y | Y | N/A | NR (in trial report) | Y |
|  |  | Rode, Klein (194) | Y | Y | Y | Y | N/A | NR (in trial report) | Y |
|  |  | Rouse, Caritis (195) | Y | Y | Y | Y | N/A | NR (in trial report) | Y |
|  |  | Senat, Porcher (196) | Y | Y | Y | Y | N/A | NR (in trial report) | Y |
|  |  | Serra, Perales (197) | Y | Y | Y | Y | N/A | NR (in trial report) | Y |
|  |  | Caritis, Rouse (198) | Y | Y | Y | Y | Y | NR (in trial report) | Y |
|  |  | Combs, Garite (199) | Y | Y | Y | Y | Y | NR (in trial report) | Y |
|  |  | Lim, Schuit (200) | Y | Y | Y | Y | N | NR (in trial report) | Y |
| Nutritional screening for improving professional practice for patient outcomes in hospital and primary care settings | Cochrane Pain, Palliative and Supportive Care Group | Moore, Siu (201) | Y | Y | N | N | N/A | NR (in trial report) | Y |

ICC=intracluster correlation coefficient; N/A=not applicable; NR=not reported; NTR=no trial report
