# Supplementary material for: Cluster Randomised Trials in Cochrane Reviews: Evaluation of Methodological and Reporting Practice
Source: PLoS One. 2016 Mar 16;11(3):e0151818. doi: 10.1371/journal.pone.0151818 (PMC4794236; doi:10.1371/journal.pone.0151818)
Supplement: S4 Table — (DOCX) [file pone.0151818.s006.docx]

**Supplementary Table 4. Assessment of “Assessing risk of bias”**

| Review Name | Cochrane Group | Is risk of recruitment bias assessed? | Is risk of baseline imbalances assessed? | Is risk of loss of clusters and individuals assessed? | Is risk of incorrect analysis assessed? | Is risk of non-comparability with individual RCTs assessed for each outcome? |
| --- | --- | --- | --- | --- | --- | --- |
| Antibiotics for preventing meningococcal infections | Cochrane Acute Respiratory Infections Group | No | No | No | No | No |
| Influenza vaccination for healthcare workers who care for people aged 60 or older living in long-term care institutions | Cochrane Acute Respiratory Infections Group | No | No | No | Yes | No |
| Integrated disease management interventions for patients with chronic obstructive pulmonary disease | Cochrane Airways Group | Yes | Yes | Yes | Yes | No |
| Physical conditioning as part of a return to work strategy to reduce sickness absence for workers with back pain | Cochrane Back Group | No | Yes | No | No | No |
| Flexible sigmoidoscopy versus faecal occult blood testing for colorectal cancer screening in asymptomatic individuals | Cochrane Colorectal Cancer Group | No | No | No | No | No |
| Mass media interventions for reducing mental health-related stigma | Cochrane Consumers and Communication Group | Yes | Yes | Yes | Yes | Yes |
| Interventions to promote informed consent for patients undergoing surgical and other invasive healthcare procedures | Cochrane Consumers and Communication Group | No | No | No | No | No |
| Enhanced care by generalists for functional somatic symptoms and disorders in primary care | Cochrane Depression, Anxiety and Neurosis Group | No | Yes | No | Yes | N/A |
| Behavioural therapies versus other psychological therapies for depression | Cochrane Depression, Anxiety and Neurosis Group | No | No | No | No | No |
| Ready-to-use therapeutic food for home-based treatment of severe acute malnutrition in children from six months to five years of age | Cochrane Developmental, Psychosocial and Learning Problems Group | Yes | Yes | Yes | No | No |
| Specially formulated foods for treating children with moderate acute malnutrition in low- and middle-income countries | Cochrane Developmental, Psychosocial and Learning Problems Group | No | Yes | No | No | No |
| Educational and skills-based interventions for preventing relationship and dating violence in adolescents and young adults | Cochrane Developmental, Psychosocial and Learning Problems Group | No | No | No | No | No |
| Non-specialist health worker interventions for the care of mental, neurological and substance-abuse disorders in low- and middle-income countries | Cochrane Effective Practice and Organisation of Care Group | No | Yes | No | Yes | No |
| Computerized advice on drug dosage to improve prescribing practice | Cochrane Effective Practice and Organisation of Care Group | No | Yes | No | No | No |
| The effect of different methods of remuneration on the behaviour of primary care dentists | Cochrane Effective Practice and Organisation of Care Group | Yes | Yes | Yes | No | N/A |
| Behavioral interventions for improving condom use for dual protection | Cochrane Fertility Regulation Group | No | No | No | No | No |
| Theory-based interventions for contraception | Cochrane Fertility Regulation Group | No | No | No | No | No |
| Remote and web 2.0 interventions for promoting physical activity | Cochrane Heart Group | No | No | No | No | No |
| Decentralising HIV treatment in lower- and middle-income countries | Cochrane HIV/AIDS Group | No | Yes | No | No | No |
| Primaquine for preventing relapse in people with *Plasmodium vivax*malaria treated with chloroquine | Cochrane Infectious Diseases Group | No | No | No | No | No |
| Mosquito larval source management for controlling malaria | Cochrane Infectious Diseases Group | No | Yes | Yes | Yes | No |
| Rifamycins (rifampicin, rifabutin and rifapentine) compared to isoniazid for preventing tuberculosis in HIV-negative people at risk of active TB | Cochrane Infectious Diseases Group | No | No | Yes | Yes | No |
| Screening for lung cancer | Cochrane Lung Cancer Group | No | Yes | No | No | No |
| Targeting intensive glycaemic control versus targeting conventional glycaemic control for type 2 diabetes mellitus | Cochrane Metabolic and Endocrine Disorders Group | No | No | No | No | No |
| Non-pharmacological interventions for fatigue in rheumatoid arthritis | Cochrane Musculoskeletal Group | No | No | No | No | No |
| Cycled light in the intensive care unit for preterm and low birth weight infants | Cochrane Neonatal Group | No | No | No | No | No |
| Enamel etching for bonding fixed orthodontic braces | Cochrane Oral Health Group | No | No | No | Yes | N/A |
| Screening programmes for the early detection and prevention of oral cancer | Cochrane Oral Health Group | No | No | No | No | N/A |
| Fluoride varnishes for preventing dental caries in children and adolescents | Cochrane Oral Health Group | No | Yes | No | Yes | No |
| Effectiveness and cost-effectiveness of home palliative care services for adults with advanced illness and their caregivers | Cochrane Pain, Palliative and Supportive Care Group | No | Yes | No | No | No |
| Interventions for implementation of thromboprophylaxis in hospitalized medical and surgical patients at risk for venous thromboembolism | Cochrane Peripheral Vascular Diseases Group | No | Yes | No | Yes | No |
| Fetal and umbilical Doppler ultrasound in high-risk pregnancies | Cochrane Pregnancy and Childbirth Group | No | Yes | Yes | No | No |
| Psychosocial interventions for supporting women to stop smoking in pregnancy | Cochrane Pregnancy and Childbirth Group | No | Yes | Yes | No | No |
| Midwife-led continuity models versus other models of care for childbearing women | Cochrane Pregnancy and Childbirth Group | No | No | No | No | No |
| Schedules for home visits in the early postpartum period | Cochrane Pregnancy and Childbirth Group | No | Yes | Yes | Yes | No |
| Telephone support for women during pregnancy and the first six weeks postpartum | Cochrane Pregnancy and Childbirth Group | No | Yes | Yes | Yes | No |
| Interventions to improve water quality and supply, sanitation and hygiene practices, and their effects on the nutritional status of children | Cochrane Public Health Group | No | Yes | No | No | No |
| User-held personalised information for routine care of people with severe mental illness | Cochrane Schizophrenia Group | No | No | No | No | No |
| Tobacco cessation interventions for young people | Cochrane Tobacco Addiction Group | No | No | No | No | No |
| Smoking cessation interventions for smokers with current or past depression | Cochrane Tobacco Addiction Group | No | No | No | No | No |
| Relapse prevention interventions for smoking cessation | Cochrane Tobacco Addiction Group | Yes | Yes | No | No | No |
| Telephone counselling for smoking cessation | Cochrane Tobacco Addiction Group | No | Yes | No | No | No |
| Nursing interventions for smoking cessation | Cochrane Tobacco Addiction Group | No | No | No | No | No |
| Internet-based interventions for smoking cessation | Cochrane Tobacco Addiction Group | No | Yes | No | No | No |
| Infection control strategies for preventing the transmission of meticillin-resistant *Staphylococcus aureus* (MRSA) in nursing homes for older people | Cochrane Wounds Group | No | Yes | No | No | N/A |
| Dressings and topical agents for preventing pressure ulcers | Cochrane Wounds Group | No | No | No | No | No |
| Interventions for cutaneous Bowen's disease | Cochrane Skin Group | No | Yes | Yes | No | No |
| Beta-lactam versus beta-lactam-aminoglycoside combination therapy in cancer patients with neutropenia | Cochrane Gynaecological Cancer Group | No | No | No | No | No |
| Prenatal administration of progesterone for preventing preterm birth in women considered to be at risk of preterm birth | Cochrane Pregnancy and Childbirth Group | No | Yes | Yes | Yes | No |
| Nutritional screening for improving professional practice for patient outcomes in hospital and primary care settings | Cochrane Pain, Palliative and Supportive Care Group | No | No | No | No | No |

N/A=not applicable; RCT=randomised controlled trial
