# Supplementary material for: Cluster Randomised Trials in Cochrane Reviews: Evaluation of Methodological and Reporting Practice
Source: PLoS One. 2016 Mar 16;11(3):e0151818. doi: 10.1371/journal.pone.0151818 (PMC4794236; doi:10.1371/journal.pone.0151818)
Supplement: S5 Table — (DOCX) [file pone.0151818.s007.docx]

**Supplementary Table 5. Assessment of “Analysing C-RCTs”**

| Review Name | Cochrane Group | Is it stated whether the reported C-RCT results are adjusted (i.e in the text, tables, or forest plots)? ^a^ | Is there a warning that CIs may be artificially narrow if unadjusted results are presented? ^b^ | Are unadjusted results from C-RCTs excluded from meta-analysis? ^c^ | Are unadjusted results adjusted using data presented in the trial reports? ^d^ | If ICC was estimated, are sensitivity analyses carried out? ^e^ | Are data from trials that adjust for clustering correctly extracted? ^f^ | Are C-RCTs and RCTs grouped in the text, tables or forest plots (to investigate heterogeneity or to allow correct interpretation)? ^g^ | Are C-RCTs grouped by unit of randomisation in the text, tables, or forest plots (to investigate heterogeneity or to allow correct interpretation)? ^h^ |
| --- | --- | --- | --- | --- | --- | --- | --- | --- | --- |
| Antibiotics for preventing meningococcal infections | Cochrane Acute Respiratory Infections Group | N | N | N | N/A | NA | N/A | N | N |
| Influenza vaccination for healthcare workers who care for people aged 60 or older living in long-term care institutions | Cochrane Acute Respiratory Infections Group | N | N/A | Y | N/A | N | Y | N | N/A |
| Integrated disease management interventions for patients with chronic obstructive pulmonary disease | Cochrane Airways Group | N | N/A | Y | N/A | N | N/A | N | N/A |
| Physical conditioning as part of a return to work strategy to reduce sickness absence for workers with back pain | Cochrane Back Group | N | N | N | N | N/A | N | N | N |
| Flexible sigmoidoscopy versus faecal occult blood testing for colorectal cancer screening in asymptomatic individuals | Cochrane Colorectal Cancer Group | Y | Y | N | N/A | N/A | N/A | N | N |
| Mass media interventions for reducing mental health-related stigma | Cochrane Consumers and Communication Group | Y | N/A | Y | Y | Y | N/A | Y | N |
| Interventions to promote informed consent for patients undergoing surgical and other invasive healthcare procedures | Cochrane Consumers and Communication Group | N | N | N | N | N/A | N/A | N | N |
| Enhanced care by generalists for functional somatic symptoms and disorders in primary care | Cochrane Depression, Anxiety and Neurosis Group | N | N/A | Y | Y | N/A | Y | N/A | N/A |
| Behavioural therapies versus other psychological therapies for depression | Cochrane Depression, Anxiety and Neurosis Group | N | N | N | N | N/A | N/A | N | N |
| Ready-to-use therapeutic food for home-based treatment of severe acute malnutrition in children from six months to five years of age | Cochrane Developmental, Psychosocial and Learning Problems Group | N | N/A | Y | Y | N | N/A | N/A | N/A |
| Specially formulated foods for treating children with moderate acute malnutrition in low- and middle-income countries | Cochrane Developmental, Psychosocial and Learning Problems Group | N | N/A | N/A | N/A | N/A | N | N | N |
| Educational and skills-based interventions for preventing relationship and dating violence in adolescents and young adults | Cochrane Developmental, Psychosocial and Learning Problems Group | N | N/A | Y | N/A | N/A | Y | N | N |
| Non-specialist health worker interventions for the care of mental, neurological and substance-abuse disorders in low- and middle-income countries | Cochrane Effective Practice and Organisation of Care Group | Y | N/A | Y | Y | N/A | Y | N | N |
| Computerized advice on drug dosage to improve prescribing practice | Cochrane Effective Practice and Organisation of Care Group | N | Y | N | N | N/A | Y | N | N |
| The effect of different methods of remuneration on the behaviour of primary care dentists | Cochrane Effective Practice and Organisation of Care Group | Y | Y | N/A | N | N/A | Y | N/A | N/A |
| Behavioral interventions for improving condom use for dual protection | Cochrane Fertility Regulation Group | Y | N | N/A | N | N/A | Y | N/A | N/A |
| Theory-based interventions for contraception | Cochrane Fertility Regulation Group | Y | N/A | N/A | N/A | N/A | Y | N/A | N/A |
| Remote and web 2.0 interventions for promoting physical activity | Cochrane Heart Group | N | N | N | N | N/A | N | N | N |
| Decentralising HIV treatment in lower- and middle-income countries | Cochrane HIV/AIDS Group | Y | N/A | Y | N/A | N | N/A | N | N |
| Primaquine for preventing relapse in people with *Plasmodium vivax*malaria treated with chloroquine | Cochrane Infectious Diseases Group | Y | N/A | N/A | N/A | N/A | Y | N | N |
| Mosquito larval source management for controlling malaria | Cochrane Infectious Diseases Group | Y | Y | N | N | Y | N/A | Y | N/A |
| Rifamycins (rifampicin, rifabutin and rifapentine) compared to isoniazid for preventing tuberculosis in HIV-negative people at risk of active TB | Cochrane Infectious Diseases Group | N | N/A | N/A | N/A | N/A | Y | N | N/A |
| Screening for lung cancer | Cochrane Lung Cancer Group | N | N | N | N | N/A | N/A | N | N/A |
| Targeting intensive glycaemic control versus targeting conventional glycaemic control for type 2 diabetes mellitus | Cochrane Metabolic and Endocrine Disorders Group | N | N/A | N/A | N/A | N/A | Y | N | N/A |
| Non-pharmacological interventions for fatigue in rheumatoid arthritis | Cochrane Musculoskeletal Group | N | N | N | N | N/A | N/A | N | N/A |
| Cycled light in the intensive care unit for preterm and low birth weight infants | Cochrane Neonatal Group | N | N | N | N | N/A | N/A | N | N/A |
| Enamel etching for bonding fixed orthodontic braces | Cochrane Oral Health Group | Y | N/A | Y | N | N/A | Y | N/A | N/A |
| Screening programmes for the early detection and prevention of oral cancer | Cochrane Oral Health Group | N | N | N/A | N | N/A | N/A | N/A | N/A |
| Fluoride varnishes for preventing dental caries in children and adolescents | Cochrane Oral Health Group | N | N/A | Y | N/A | Y | Y | N | N |
| Effectiveness and cost-effectiveness of home palliative care services for adults with advanced illness and their caregivers | Cochrane Pain, Palliative and Supportive Care Group | N | N | Y | N/A | N | Y | N | N |
| Interventions for implementation of thromboprophylaxis in hospitalized medical and surgical patients at risk for venous thromboembolism | Cochrane Peripheral Vascular Diseases Group | N | Y | N | N/A | N/A | N | N | N |
| Fetal and umbilical Doppler ultrasound in high-risk pregnancies | Cochrane Pregnancy and Childbirth Group | N | Y | N | N/A | N/A | N/A | Y | N/A |
| Psychosocial interventions for supporting women to stop smoking in pregnancy | Cochrane Pregnancy and Childbirth Group | N | N/A | Y | Y | Y | Y | N | N |
| Midwife-led continuity models versus other models of care for childbearing women | Cochrane Pregnancy and Childbirth Group | N | N/A | N/A | N/A | N/A | Y | N | N/A |
| Schedules for home visits in the early postpartum period | Cochrane Pregnancy and Childbirth Group | N | N/A | N/A | N/A | N/A | Y | N | N |
| Telephone support for women during pregnancy and the first six weeks postpartum | Cochrane Pregnancy and Childbirth Group | Y | N/A | N/A | N/A | N/A | Y | Y | N/A |
| Interventions to improve water quality and supply, sanitation and hygiene practices, and their effects on the nutritional status of children | Cochrane Public Health Group | N | N/A | Y | Y | N/A | Y | N | N |
| User-held personalised information for routine care of people with severe mental illness | Cochrane Schizophrenia Group | N | Y | N | N/A | Y | N | N | N/A |
| Tobacco cessation interventions for young people | Cochrane Tobacco Addiction Group | N | N | N | N | N/A | N | N | N |
| Smoking cessation interventions for smokers with current or past depression | Cochrane Tobacco Addiction Group | N | N/A | N/A | N/A | N/A | N | N/A | N/A |
| Relapse prevention interventions for smoking cessation | Cochrane Tobacco Addiction Group | N | N | N | N | N/A | Y | N | N |
| Telephone counselling for smoking cessation | Cochrane Tobacco Addiction Group | N | N | N | N | N/A | N | N | N |
| Nursing interventions for smoking cessation | Cochrane Tobacco Addiction Group | N | N | N | N | N/A | N | N | N/A |
| Internet-based interventions for smoking cessation | Cochrane Tobacco Addiction Group | N | N/A | N/A | N/A | N/A | N/A | N/A | N/A |
| Infection control strategies for preventing the transmission of meticillin-resistant *Staphylococcus aureus* (MRSA) in nursing homes for older people | Cochrane Wounds Group | Y | N/A | N/A | N/A | N/A | Y | N/A | N/A |
| Dressings and topical agents for preventing pressure ulcers | Cochrane Wounds Group | Y | N | N/A | N | N/A | N/A | N | N/A |
| Interventions for cutaneous Bowen's disease | Cochrane Skin Group | N | Y | N | N | N/A | N/A | N | N/A |
| Beta-lactam versus beta-lactam-aminoglycoside combination therapy in cancer patients with neutropenia | Cochrane Gynaecological Cancer Group | N | N | N | N | N/A | NTRs | N | N/A |
| Prenatal administration of progesterone for preventing preterm birth in women considered to be at risk of preterm birth | Cochrane Pregnancy and Childbirth Group | N | Y | N | N/A | N/A | N | Y | N/A |
| Nutritional screening for improving professional practice for patient outcomes in hospital and primary care settings | Cochrane Pain, Palliative and Supportive Care Group | N | N | N/A | N | N/A | N/A | N | N/A |
| ^a^ Criteria was applicable to all reviews  ^b^ Criteria was applicable for 27 reviews that presented unadjusted results  ^c^ Criteria was applicable for 34 reviews where data from unadjusted C-RCTs was eligible for inclusion in the conducted meta-analyses  ^d^ Criteria was applicable for 27 reviews that included trials with unadjusted data, and that did not state that it would not be possible to adjust data themselves  ^e^ Criteria was applicable for 10 reviews that estimated an ICC to adjust cluster data  ^f^ Criteria was applicable for 31 reviews where included trials reported adjusted data  ^g^ Criteria was applicable for 40 reviews that included both C-RCTs and I-RCTs in the same analysis  ^h^ Criteria was applicable to 22 reviews that included C-RCTs with different units of randomisation in the same analysis | | | | | | | | | |

C-RCT=cluster-randomised controlled trial; ICC=intracluster correlation coefficient; N/A=not applicable; NTRs=no trial reports; RCT=randomised controlled trial
