# Supplementary material for: Cluster Randomised Trials in Cochrane Reviews: Evaluation of Methodological and Reporting Practice
Source: PLoS One. 2016 Mar 16;11(3):e0151818. doi: 10.1371/journal.pone.0151818 (PMC4794236; doi:10.1371/journal.pone.0151818)
Supplement: S6 Table — (DOCX) [file pone.0151818.s008.docx]

**Supplementary Table 6. Additional results for “Reporting C-RCTs”**

| **Reporting C-RCTs** | **Assessment** |
| --- | --- |
| Is the unit of randomisation reported for each C-RCT? | 44/49* (89.8%) reported for all C-RCTs  2/49* (4.1%) reported for at least one C-RCT |
| Is the study design (i.e. matched pairs, stratified) reported for each C-RCT? | 34/49* (69.4%) reported for all C-RCTs  10/49* (20.4%) reported for at least one C-RCT |
| Is it reported whether the trial is adjusted or unadjusted for clustering for each C-RCT for each outcome? | 29/49* (59.2%) reported for all C-RCTs  9/49* (18.4%) reported for at least one C-RCT |
| Is the method of cluster adjustment reported for each C-RCT for each outcome? | 8/33^$^ (24.3) reported for all C-RCTs  4/33^$^ (12.1) reported for at least one C-RCT |
| Is the ICC reported for each C-RCT for each outcome? | 7/23^£^ (30.4%) reported for all C-RCTs  6/23^£^ (26.1%) reported for at least one C-RCT |
| Is the average cluster size reported for each C-RCT? | 22/46^&^ (47.8%) reported for all C-RCTs  12/46^&^ (26.1%) reported for at least one C-RCT |
| * 1 review excluded as none of the trial reports for the included C-RCTs could be obtained via inter-library loans  ^$^ 17 reviews excluded (1 review: none of the trial reports for the included C-RCTs could be obtained; 4 reviews: as all trials did not report method of adjustment; 12 reviews: as all trials were unadjusted  ^£^ 27 reviews excluded (1 review: none of the trial reports for the included C-RCTs could be obtained; 23 reviews: all trials did not report ICC, 3 reviews: some trials did not report ICC and some trial reports were unavailable)  ^&^ 4 reviews excluded (1 review: none of the trial reports for the included C-RCTs could be obtained; 3 reviews: all trials did not report average cluster size) | |
